# Mitofusin-2 modulates the epithelial to mesenchymal transition in thyroid cancer progression

**DOI:** 10.1038/s41598-021-81469-0

**Published:** 2021-01-21

**Authors:** Mi-Hyeon You, Min Ji Jeon, Seong ryeong Kim, Woo Kyung Lee, Sheue-yann Cheng, Goo Jang, Tae Yong Kim, Won Bae Kim, Young Kee Shong, Won Gu Kim

**Affiliations:** 1 Department of Internal Medicine, Asan Medical Center, University of Ulsan College of Medicine, 88, Olympic-ro 43-gil, Songpa-gu, Seoul, 05505 South Korea; 2 College of Veterinary Medicine, Seoul National University, 1 Gwanak-ro, Gwanak-gu, Seoul, 08826 South Korea; 3 Laboratory of Molecular Biology, Center for Cancer Research, National Cancer Institute, National Institutes of Health, 37 Convent Dr., Bethesda, MD 20892-4264 USA

**Keywords:** Cancer, Endocrinology

## Abstract

Here, we investigated the potential roles of Mitofusin-2 (MFN2) in thyroid cancer progression. MFN2 regulates mitochondrial fusion/division in cells and plays an important role in various aspects of cell metabolism. MFN2 might involve in cell cycle regulation, apoptosis, and differentiation, and it might play a role as a tumor suppressor in carcinogenesis. We evaluated the prognostic impacts of *MFN2* expression in thyroid cancer by analyzing TCGA data. In vitro and in vivo, MFN2 was knocked out using CRISPR/Cas9 or siRNA, and MFN2 was stably overexpressed in two thyroid cancer cell lines (Cal62 and HTH83). TCGA analysis revealed that *MFN2* expression was lower in thyroid cancer than in normal tissues and significantly associated with a degree of differentiation, *RAS* mutations, and less lymph node metastasis. *MFN2* expression was significantly correlated with cell adhesion molecules and epithelial to mesenchymal transition (EMT) in a gene-set enrichment assay. MFN2 knock-out (KO) in Cal62 and HTH83 cells using CRISPR/Cas9 or siRNA significantly promoted cell migration and invasion in vitro. The same trends were observed in MFN2 KO mouse embryonic fibroblasts (MEFs) compared to those in the controls (MFN2 WT MEFs). Conversely, MFN2 overexpression in cancer cell lines greatly inhibited cell migration and invasion. However, there was no difference in colony formation and proliferation in Cal62 and HTH83 cells after modulating MFN2, although there were significant differences between MFN KO and WT MEFs. EMT-associated protein expression was induced after MFN2 KO in both cancer cell lines. The mechanistic results suggest that MFN2 might modulate EMT through inducing the AKT signaling pathway. EMT-associated changes in protein expression were also confirmed by modulating MFN2 in xenograft tumors. Thus, MFN2 acts as a tumor suppressor in thyroid cancer progression and metastasis by modulating EMT.

## Introduction

Mitochondria are essential organelles that produce ATP, reactive oxygen species^1^, redox molecules, and metabolites and regulate cell signaling and biosynthetic metabolism. They sense stresses and allow for cellular adaptation to the microenvironement^2,3^. For these reasons, mitochondrial reprogramming is important during carcinogenesis and cancer progression. Some studies have evaluated the role of mitochondrial alterations in thyroid cancer. Because high levels of ROS are produced during thyroid hormone synthesis, mitochondrial defense mechanisms against ROS are important in thyroid follicular cells. These antioxidant defense systems have been found to be inactive in thyroid cancers^4,5^. In Hürthle cell carcinoma, a rare type of differentiated thyroid cancer is characterized by excess mitochondria as well as defective mitochondrial oxidative phosphorylation^6^. One study also reported that the subcellular localization of the BRAF^V600E^ mutant in mitochondria was associated with a suppressed apoptotic response, resistance to RAF inhibitors, and reduced mitochondrial oxidative phosphorylation in thyroid cancer^7^

Mitofusin-2 (MFN2) is a conserved dynamin-like GTPase located on the outer membrane of mitochondria. MFN2 in conjunction with Mitofusin-1 located on the inner membrane regulates mitochondrial fusion/fission, and is known to play an important role in various aspects of cell metabolism^8,9^. The congenital *MFN2* mutation causes Charcot-Marie-Tooth 2A (CMT2A) disease, a genetic disorder characterized by neuromuscular dysfunction that results in gradual skeletal muscle loss^1,10^. Recently, MFN2 has also been shown to be an important regulator of cancer progression. Studies on breast, pancreatic, and lung cancer demonstrated that MFN2 inhibits cell proliferation or cancer progression through interact with RAS or suppression of phosphoinositide 3 kinase (PI3K)-protein kinase B (AKT) signaling^11–14^. MFN2 is also involved in cell cycle regulation, apoptosis, and differentiation, and it might play a role in cancer development^15,16^. However, to our knowledge, currently there are no studies on the role of MFN2 in thyroid cancer.

In this study, we evaluated the potential roles of MFN2 in the progression of thyroid cancer. First, we analyzed the expression of *MFN2* in human thyroid cancer using data from The Cancer Genome Atlas (TCGA)^17^. The expression of *MFN*2 was relatively higher in normal thyroid tissues than that in cancer tissues. The relatively higher expression of *MFN2* was significantly associated with well-differentiated tumors, *RAS* mutations, and lower incidence of cervical lymph node (LN) metastasis. The gene set enrichment assay suggested that *MFN2* expression was associated with cell adhesion molecules and epithelial-mesenchymal transition (EMT). Therefore, we evaluated the role of MFN2 as a tumor suppressor in thyroid cancer progression in the context of EMT.

## Materials and methods

### Analysis of TCGA data

A transcriptome thyroid cancer dataset from TCGA (TCGA-THCA, http://cancergenome.nih.gov/; normal thyroid tissue [n = 59] and thyroid cancer [n = 505]) was used to investigate the clinical significance of *MFN2*. For this, we analyzed the transcriptome data of tumors with *MFN2* expression in the lower and upper quartiles; low (n = 126) and high (n = 126) *MFN2* tumor groups. To evaluate the biological characteristics of tumors based on *MFN2* expression, we applied thyroid differentiation scores (TDS) and BRAFV600E-RAS scores (BRS) to the TCGA data as previously reported^18^. Lower TDS indicate less-differentiated tumors (like poorly differentiated or anaplastic thyroid cancer), and negative and positive BRS indicate BRAFV600E-like and RAS-like tumors, respectively. We also compared *MFN2* expression between low (n = 97 in the lower quartiles) and high (n = 97 in the upper quartiles) TDS and BRS tumor groups which were divided according to TDS and BRS, respectively. To determine the relationship between *MFN2* expression and tumor invasiveness, we performed gene set enrichment analysis (GSEA, http://software.broadinstitute.org/gsea/index.jsp) for KEGG or Hallmark gene sets between the two groups based on *MFN2* expression. In our settings, a negative normalized enrichment score (NES) indicated gene set enrichment in the ranked list for the *MFN2* low tumor group. In these ranked gene sets, those with a nominal *p* value < 0.05 and an FDR q-value < 0.05 were considered significant.

### Cell culture

Cal62 (*KRAS* mutation) and HTH83 (*HRAS* mutation) cells derived from anaplastic thyroid cancer (ATC) with a *RAS* mutation and mouse embryonic fibroblasts (MEFs) (both wild-type [WT] and *MFN2* knockout [KO]) were used in the experiments. Cal62 and HTH83 cells were purchased from the German Collection of Microorganisms and Cell Cultures (DSMZ, Germany), and MEFs (both WT and KO) were purchased from the American Type Culture Collection (ATCC, USA). These cell lines were verified by short tandem repeat profiling. All these cell lines were maintained in DMEM (GIBCO, Grand Island, NY, USA) supplemented with 10% fetal bovine serum at 37 ℃ in 5% CO_2_. Media were changed every 2‒3 days, and sub-culturing was performed when the cells reached approximately 80% confluency.

### Overexpression of *MFN2*

To overexpress *MFN2*, the promoter (Elongation factor 1 α, EF1α) and cDNA (MFN2) tagged with the Flag epitope were cloned into the piggyBac (PB) transposon backbone, which contains green fluorescent protein (GFP) under the control of the EF1α promoter. One microgram of the vector and transposase was transfected into Cal62 cells using an electroporation protocol. Only GFP-positive cells were manually selected and further cultured in DMEM (GIBCO) supplemented with 10% FBS and 1% antibiotics.

### Knockout of *MFN2*

To KO *MFN2*, single-guided (sg) RNA sequences were designed using “CHOPCHOP” web-based software and synthesized with the GeneArt Precision gRNA Synthesis Kit (Invitrogen, San Jose, CA, USA). Cas9 protein (TrueCut Cas9 Protein v2, Invitrogen) and sgRNA were transfected into Cal62 cells using an electroporation device (Program #16, Neon, Invitrogen). After 48 h of transfection, one-half of the cells were used to assess the mutation using the T7E1 assay, and the other half of the cells was used for single cell colony culture. Each single cell colony was cultured and analyzed using the T7E1 assay to verify the KO.

### T7E1 assay

Genomic DNA was extracted using a DNA Extraction Kit (DNeasy Blood & Tissue Kit, QLAGEN) after 2 days of transfection. CRISPR-Cas9 target sites were amplified using primer pairs (Exon 4, Forward: GAACCAGCTTCAGAACCAGGC and Reverse: GCTGGAAGCTCATTACAGCC; Exon 5, Forward: GGAGACAGACAGACAGTCTC and Reverse: CTGTGGAGGTGAATGGGAGAC) by PCR. The PCR was progressed under the condition (94 °C for 5 min, 35–40 cycles of 94 °C for 20 s/57 °C for 30 s/72 °C for 35 s, and 72 °C for 5 min). T7E1 analysis was performed. The amplicons were denatured by heating and annealed to form heteroduplex DNA, which was treated with 5 units of T7 endonuclease 1 (Toolgen Inc., Seoul, Korea) for 20 min at 37 °C and then analyzed by electrophoresis on a 1.5% agarose gel.

### Small interfering RNA transfection

Four different MFN2 small interfering RNAs (si-RNAs) against human MFN2 or scrambled si-RNAs were transiently transfected into HTH83 cells using Lipofectamine 3000 Reagent (Invitrogen, Carlsbad, CA, USA) according to the manufacturer’s instructions^19^. In brief, cells were seeded in 6-well plates at 70–80% confluence when transfected. Cells of each well were transfected with 10 μl of 20 μM siRNA was added to 125 μl of Opti-MEM (GIBCO) with P3000 (4 μl) was added to 125 μl of Opti-MEM with 3.75 μl of Lipofectamine 3000 Reagent for 5 min. Mix gently and incubated for 12 min at room temperature, about 250 μl of the transfection mixture was added to cells. After 12 h transfection, the medium was changed by new media.

SiRNAs specific for MFN2 and scrambled si-RNAs were purchased from DHARMACON (Lafayette, CO, USA) and were described as follows:

- Human MFN2-#1, ACUAUAAGCUGCGAAUUAA;
- Human MFN2-#2, GAUCAGGCGCCUCUCUGUA;
- Human MFN2-#3, GGUUACCUAUCCAAAGUGA;
- Human MFN2-#4, CAACUAUGACCUAAACUGU.

And those for the scrambled si-RNAs were as follows:

- Human Control-#1, UAGCGACUAAACACAUCA;
- Human Control-#2, UAAGGCUAUGAAGAGAUAC;
- Human Control-#3, AUGUAUUGGCCUGUAUUAG;
- Human Control-#4, AUGAACGUGAAUUGCUCAA.

The scrambled si-RNAs did not match any human sequences in a Gene Bank search.

### Cell proliferation assay

The effects of MFN2 KO or overexpression on Cal62 and HTH83 cell proliferation were evaluated by 3-(4, 5-dimethylthiazol-2-yl)-2, 5-diphenyltetrazolium bromide assay (MTT assay; Cell Bio labs, San Diego, CA, USA)^19^. Cells were seeded in 24 or 96 well plated for different durations (1, 2, 3, 4, and 5 days after stable transfection). Then, Cell proliferation was measured with using the MTT assay as previously described^19^.

### Colony forming assay

Each experimental and control group cell were seeded in six well plates at a density of 5 × 10^3^ cells per well in 2 ml of media. Media were changed after 24 h. Cells were incubated for 7 days before counting. Then, the cells on the plate were then fixed and stained with 0.1% crystal violet in 20% methanol. Statistical significance was investigated by counting the number of colonies.

### Matrigel invasion assay

Invasion ability of each experimental and control group cells were examined using Matrigel Invasion Chambers (BD Bioscience). After Matrigel coating for 1 h at 37 °C for gel formation, 10^5^ cells (Cal62 cells, HTH83 cells, and MEFs) in serum-free medium were placed in the coated upper chamber in serum-free medium. Then, normal medium containing 10% FBS was added to the lower chamber. After 24 h, the cells remaining on the upper membrane were completely removed with a cotton swab, and the cells invading through the membrane were counted by staining with 20% methanol and 0.1% crystal violet.

### Migration assay

Cells were seeded in 6- well plates and a wound was artificially scraped off with a pipette tip after 9–24 h as previously described^19^. Briefly, after initial scratching, cells were washed with phosphate buffered saline (PBS), photographed, and changed with normal medium. Wound closure was observed during various times (after 6, 9, 12, 24 h), the image was photographed using a microscope. At least three measurements were taken and average measurement was calculated using IMAGE J2 (https://imagej.net).

### Western blotting

First, 30–50 μg of whole cell lysate from mouse and human samples was resolved on NuPAGE gels (Invitrogen, Thermo Fisher Scientific, Waltham, MA, USA) and transferred to 0.45 μm nitrocellulose membranes (Amersham Bioscience, Piscataway, NJ, USA). Membranes were blocked in 5% bovine serum albumin (BSA) prepared in tri-buffered saline containing 0.1% Tween 20 (TBS-T) and then incubated with specific antibodies overnight at 4 °C. The membranes were washed with TBS-T and incubated with horseradish peroxidase-conjugated secondary antibody for 1 h, at Room temperature. After washing with TBS-T, immunoreactive proteins were detected using enhanced chemiluminescence (Enzo Life Sciences, New York, USA). Anti-MFN2 (Abcam, ab56889; Cell Signaling, 9483), PathScan Multiplex Western Cocktail I (Cell Signaling, 5301), anti-Vimentin (Cell Signaling, 5741), anti-neural cadherin (N-cadherin; Cell Signaling, 13116), anti-SNAIL (Cell Signaling, 3879), anti-ZEB1 (TCF8; Cell Signaling, 3396), anti-epithelial cadherin (E-cadherin; Cell Signaling, 3195), anti-glyceraldehyde-e-phosphate dehydrogenase (GAPDH; Abcam, ab8245), and β-actin (Cell Signaling, 4970) were used. The expression of the experimental proteins was normalized to that of GAPDH or β-actin in each sample.

All methods were carried out in accordance with relevant guidelines and regulations.

### In vivo mouse xenograft study

All animal procedures and protocols in this study were approved by the Institutional Animal Care and Use Committee of the Asan Institute of Life Sciences, Seoul, Korea. Five-week-old female athymic Balb/c nude mice (OrientBio, Korea) were used. Cells (Control Cal62 Flag-vector and Flag-MFN2 cells) were prepared, and 1 × 10^6^ cells in 200 μl of suspension mixture with Matrigel (BD Biosciences) were subcutaneously inoculated into the right flank of the mice (eight mice each group). Body weight and tumor size were monitored twice weekly. Tumor size was measured using calipers, and tumor volume was calculated as (length × width^2^)/2. Tumor weight was determined after dissection of tumor tissues from euthanized mice at the study end-point.

### Reagents

Media and cell culture reagents were purchased from GIBCO.

### Statistics

Categorical variables are presented as numbers and percentages, and continuous variables are expressed as the mean with SD or median with inter-quartile range. Comparisons of continuous variables were performed using Student’s *t*-test. Comparisons between each group based on categorical variables were performed using Fisher’s exact test. To examine the relationship of *MFN2* gene expression with TDS and BRS in the TCGA-THCA, we used Pearson correlation coefficient for the analyses. Significant difference between three or more variables was analyzed using one-way ANOVA or two-way ANOVA (in vivo mouse xenograft models). Data were analyzed using SPSS statistics version 19.0 (SPSS Inc.). All *P* values were two sided, with *P* < 0.05 considered to be significant. GraphPad Prism version 5.01 (GraphPad Software INC.,San Diego, CA, USA) was used to construct the graphs.

The study was carried out in compliance with the ARRIVE guidelines.

## Results

### Expression of *MFN2* in thyroid cancer

**Figure 1:**
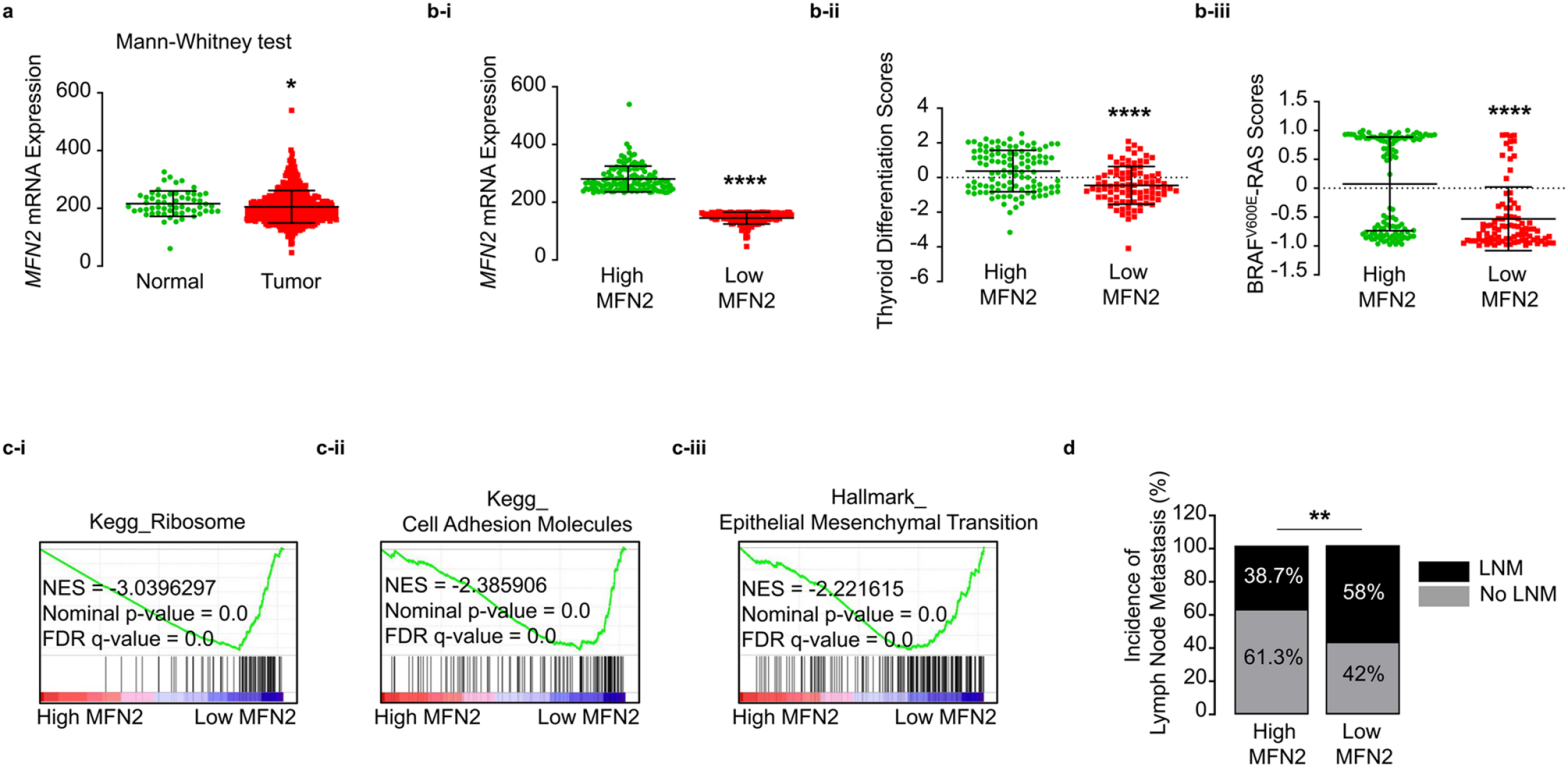
*MFN2* expression is a prognostic marker in human thyroid cancer. (**a**) Comparison of the mRNA expression of *MFN2* between normal thyroid tissue (n = 59) and tumors (n = 505). (**b**) Comparison of *MFN2* expression (**i**), thyroid differentiation scores (**ii**), and BRAFV600E-RAS scores (**iii**) between high and low *MFN2* tumor groups (n = 126 for both). (**c**) Tumor invasion-associated gene sets enriched in the low *MFN2* tumor group compared to those enriched in the high *MFN2* group (n = 126 for both). (**d**) Comparison of the incidence of cervical lymph node metastasis between the high and low *MFN2* tumor groups (n = 126 for both). (A-D) TCGA-THCA data analyses. Data represent the mean ± SD. Asterisks (p < 0.05 [*], p < 0.01 [**], p < 0.0001 [****]) indicate significant differences from the statistical analyses. *THCA* thyroid carcinoma.

To determine the clinical significance of MFN2 in human thyroid cancer, we performed an integrative analysis using TCGA-THCA data. We first found that the expression of *MFN2* was significantly lower in tumors than that in normal thyroid tissues (p < 0.05, Fig. 1a). To analyze the biological characteristics of tumors in terms of *MFN2* expression, we stratified TCGA data based on *MFN2* expression as previously described into low and high MFN2 tumor groups (Fig. 1b-i). Notably, high MFN2 tumors exhibited higher TDS p < 0.001, Fig. 1b-ii) and higher BRS (p < 0.001, Fig. 1b-iii), indicating that these tumors were well differentiated and contained *RAS* mutations rather than *BRAF* mutations. We also confirmed that TDS (Supplementary Fig. 1A-i) and BRS (Supplementary Fig. 1B-i) had positive correlation with *MFN2* mRNA expression, and well differentiated tumors (high TDS tumors, Supplementary Fig. 1A-ii) and RAS-like tumors (high BRS tumors, Supplementary Fig. 1B-ii) showed significantly higher *MFN2* expression than poorly differentiated (low TDS tumors) and BRAFV600E-like tumors (low BRS tumors), respectively. Interestingly, our GSEA between the two groups clearly indicated that the enrichment of components involved in tumor invasion such as ribosomes (Fig. 1c-i), cell adhesion molecules (Fig. 1c-ii), and EMT (Fig. 1c-iii) significantly differed based on MFN2 expression. Concordantly, the incidence of cervical LN metastases was significantly lower in the high MFN2 tumor group compare to low MFN2 tumor group (p < 0.05, Fig. 1d). *MFN2* expression was significantly higher in no LN metastasis group than LN metastasis group (Supplementary Fig. 1C). Taken together, these results suggest that MFN2 plays an important role in thyroid cancer progression, and that low *MFN2* expression is significantly associated with tumor aggressiveness in human thyroid cancer.

### Mitofusin-2 modulates cell invasion and migration in thyroid cancer cells

Based on TGCA data, the effects of *MFN2* expression were investigated using Cal62 and HTH83 cells. We investigated the changes in cancer cell proliferation, migration, and invasion after modulating the expression of *MFN2*. The expression of MFN2 was successfully downregulated in Cal62 cells using the CRISPR/Cas9 KO method (Supplementary Fig. 2). Stable overexpression of *MFN2* in the Cal62 and HTH83 cell lines was also confirmed by western blotting and GFP fluorescence (Supplementary Fig. 2).

There was no significant difference in cell proliferation after KO or overexpression of *MFN2* in these two thyroid cancer cell lines (Supplementary Fig. 3A,B). However, in MEFs, proliferation was significantly higher in the *MFN2* KO group than that in WT cells, which was consistent with the results of other studies (Supplementary Fig. 4A,B)^14^.

**Figure 2:**
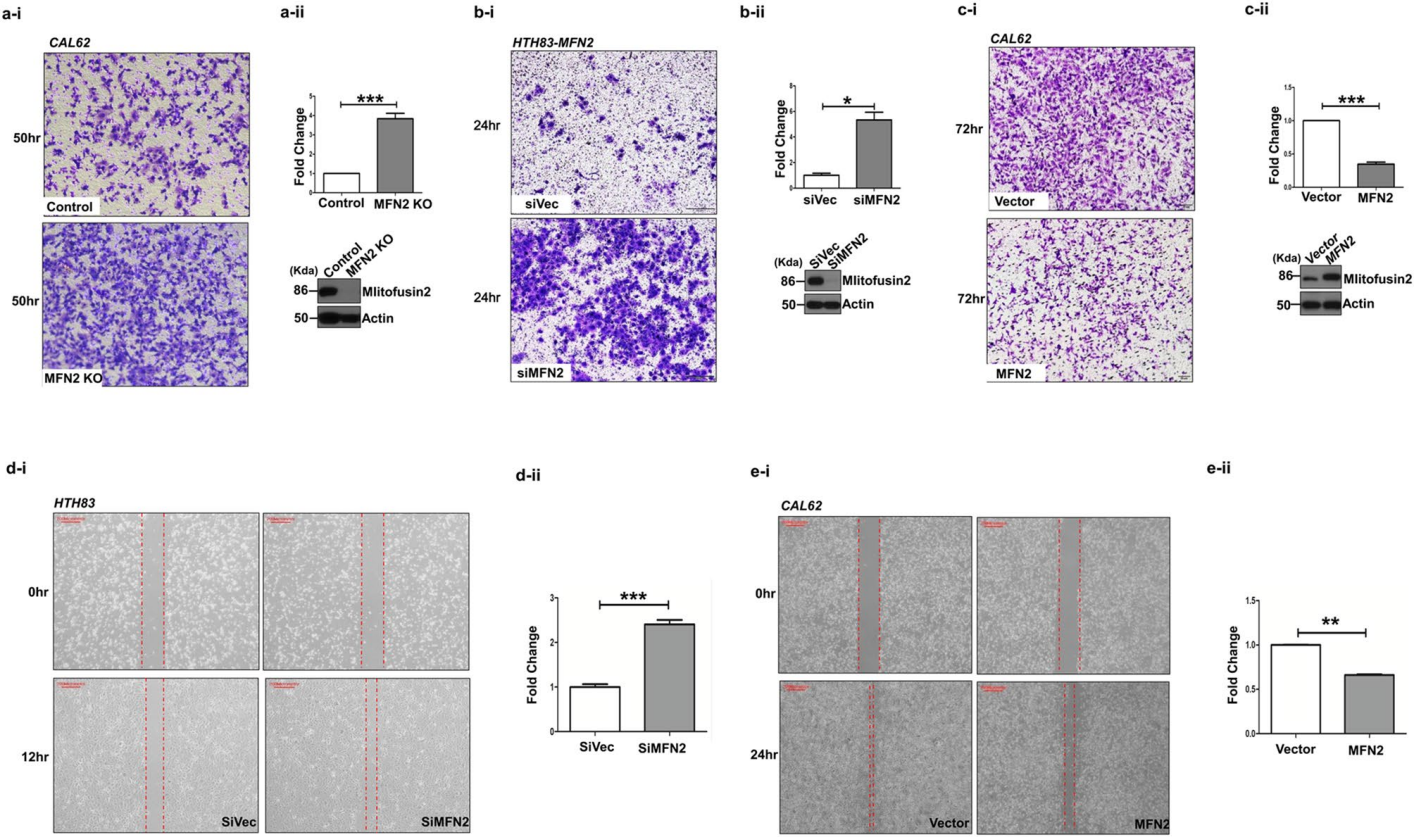
Mitofusin-2 inhibits migration and invasion ability of Cal62 and HTH83 cancer cells. (**a-i**) Transwell assays were performed in the Cal62 control and *MFN2* KO groups. A representative image was obtained from an image dyed 50 h after the initial seeding (pore size 0.4 μm). The image was acquired at a magnification of ×100; scale bar represents 50 μm. (**a-ii**) Graph of the quantified results of (**a-i**). Significantly more cells migrated in the *MFN2* KO group than those in the control group. (**b-i**) Transwell assays were performed in the HTH83 siVector and siMFN2 groups. A representative image was acquired 24 h after the initial seeding (pore size 0.8 μm). The image was acquired at a magnification of ×100; scale bar represents 50 μm. (**b-ii**) Graph of the quantified results of (**b-i**). Significantly more cells migrated in the siMFN2 group (HTH83 *MFN2* cell line) than those in the si Vector group. (**c-i**) Transwell assays were also performed in the Cal62 Vector and *MFN2* overexpression groups. A representative image was acquired 72 h after the initial seeding (pore size 0.4 μm). The image was acquired at a magnification of ×100; scale bar represents 50 μm. (**c-ii**) Graph of the quantified results of (**c-i**). Significantly fewer cells migrated in the MFN2 overexpression group than those in the control group. (**d-i**) Wound-healing assays were also performed in the HTH83 siVector and HTH83 siMFN2 groups using the HTH83 *MFN2* cell line. We measured the gap distance 12 h after the initial scratching. (**d-ii**) Quantification of the results of (**d-i**) compared to the initial scratch size of the first gap distance after 12 h. (**e-i**) Wound-healing assays were performed to investigate the differences between the Cal62 control and Cal62 *MFN2* KO groups. We measured the gap distance 9 h after the initial scratching. (**e-ii**) Quantification of the results of (**e-i**) compared to the initial scratch size of the first gap distance after 9 h. Asterisks (p < 0.05 [*], p < 0.01 [**], p < 0.001 [***]) indicate significant differences in the statistical analyses. Each data point represents the mean ± standard error of three independent experiments.

A significant increase in Cal62 cancer cell invasion was observed after *MFN2* KO compared with that in the controls (*p* < 0.001, Fig. 2a-i,a-ii). Cancer cell invasion was also significantly increased in si*MFN2*-HTH83 cells compared with that in siVec-HTH83 cells (*p* < 0.05, Fig. 2b-i,b-ii). The levels of MFN2 protein were also successfully decreased in HTH83 cells using the siRNA method (Fig. 2b-ii). Conversely, cancer cell invasion was significantly reduced in *MFN2*-overexpressing Cal62 cells compared with that in control Cal62 cells (*p* < 0.001, Fig. 2c-i,c-ii). The inhibition of cancer cell invasion by *MFN2* overexpression was also confirmed in HTH83 cells (*p* < 0.001, Supplementary Fig. 5A). We also evaluated cancer cell migration after modulation of *MFN2* in these two cancer cell lines. Cancer cell migration was significantly accelerated in HTH83 cells when *MFN2* expression was suppressed by siRNA (*p* < 0.001, Fig. 2d-i,d-ii). Cell migration was significantly decreased in MFN2-overexpressing cells compared with that in the controls for Cal62 cells (*p* < 0.01, Fig. 2e-i,e-ii) and HTH83 cells (*p* < 0.01, Supplementary Fig. 5B). Invasion and migration assays were also performed using the MFN2-WT MEFs and MFN2-KO MEFs, and there was a significant increase in invasion and migration in the MFN2-KO group compared to that in the WT group (*p* < 0.01, Supplementary Fig. 6A,B). These results indicate that the MFN2 regulates cell invasion and migration in thyroid cancer cells as well as in fibroblasts.

### Mitofusin-2 modulates EMT in thyroid cancer cells

**Figure 3:**
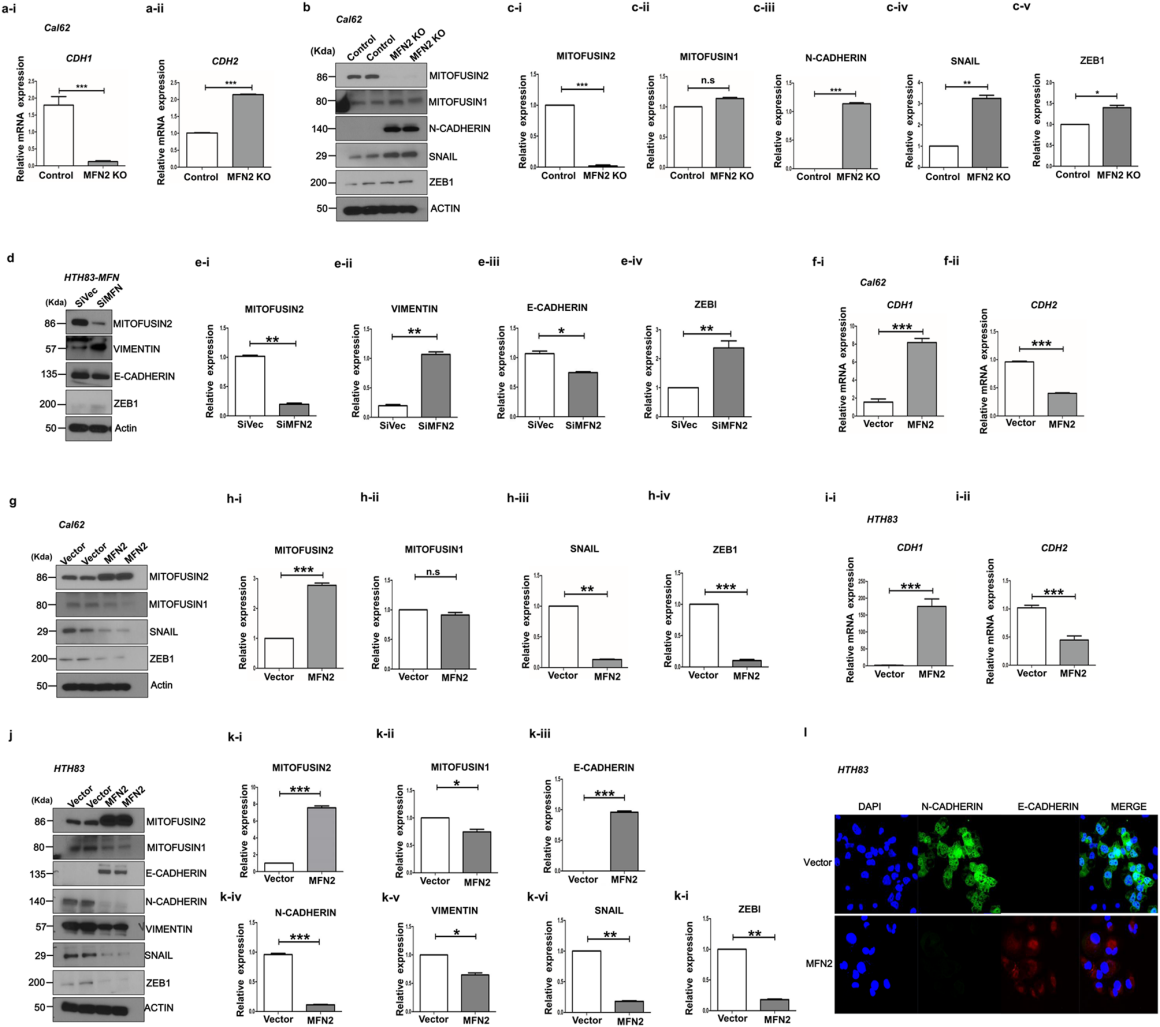
Mitofusin-2 regulates EMT in Cal62 and HTH83 cells. Effects of the knock-out or overexpression of MFN2 in Cal62 cell lines. (**a**) qRT-PCR analysis of *CDH1* (**a-i**) and *CDH2* (**a-ii**) between the Cal62 Control and MFN2 KO groups. 18S was used as an internal control. (**b**) Cal62 Control and MFN KO cell line (20 µg) lysates, and then, western blotting was performed to examine the expression of MITOFUSIN2, MITOFUSIN1, N-cadherin, SNAIL, and ZEB1. Actin was used as an internal control. (**c**) Graph of the quantified results of (**b**). (**c-i**) The expression of MITOFUSIN2 was significantly reduced. (**c-ii**) Complementarily, it was confirmed that the expression of MITOFUSIN1 was slightly increased (n.s; no significant difference). (**c-iii**) N-Cadherin expression was significantly increased in the *MFN2* KO group compared to that in the Control group. (**c-iv**, **v**) The expression of SNAIL and ZEB1 in the *MFN2* KO group was significantly increased compared to that in the Control group. (**d**) Lysates were prepared using HTH83 cell line overexpressing *MFN2* (20 μg), and western blotting was performed to examine the expression of MITOFUSIN2, VIEMNTIN, E-cadherin, and ZEB1. Actin was used as an internal control. (**e**) Graph of the quantified results of (**d**). The expression of MFN2 was significantly increased compared to that in the control (Vector) (**e-i**). (**e-ii**) The expression of VIMENTIN was significantly increased in the MITOFUSIN2 overexpression group compared to that in the control group. (**e-iii**) The expression of E-cadherin was significantly increased in the *MFN2* overexpression group compared to that in the control group. (**e-iv**) Compared with that in the control group, the expression of ZEB1 in the *MFN2* overexpression group was significantly increased. (**f**, **g**) Effects of the overexpression of MFN2 in the Cal62 cell line on the expression of EMT-related genes (mRNA, protein). (**f**) qRT-PCR analysis of *CDH1* (**f-i**) and *CDH2* (**f-ii**) between the Cal62 Vector and Cal62 *MFN2* groups. 18S was used as an internal control. (**g**) Immunoblotting for the expression of MITOFUSIN2, MITOFUSIN1, SNAIL, and ZEB1 in the Cal62-Vector and MFN2 overexpression groups. (**h-i**–**iv**) Quantification of (**g**). (**h-i**) MITOFUSIN2 in the MFN2 group was significantly increased compared with that in the Vector group. (**h-ii**) Complementarily, it was confirmed that the expression of MITOFUSIN1 was slightly decreased (n.s; no significant difference). (**h-iii**) The expression of SNAIL in the MFN2 group was significantly increased compared with that in the Vector group. (**h-iv**) The expression of ZEB1 in the MFN2 group was also significantly increased compared with that in the Vector group. (**i**–**k**) Effects of *MFN2* overexpression in the HTH83 cell line on the expression of EMT-related genes (mRNA, protein). (**i**) qRT-PCR for *CDH1* (**i-i**) and *CDH2* (**i-ii**) expression between the HTH83 Vector and MFN2 groups. (**j**) Immunoblotting for investigating the expression of MITOFUSIN2, MITOFUSIN1, E-cadherin, N-cadherin, VIMENTIN, SNAIL, and ZEB1 in the HTH83-*MFN2*-siVector and siMFN2 groups. (**k-i**–**iv**) Quantification of (**j**). The (**k-i**) expression of MITOFUSIN2 was significantly increased. (**k-ii**) Complementarily, the expression of MITOFUSIN1 was slightly decreased. (**k-iii**) The expression of E-cadherin was significantly increased in the *MFN2* group compared to that in the Control group. (**k-iv**, **v**) The expression of N-cadherin and VIMENTIN was significantly decreased. The expression of SNAIL and ZEB1 in the *MFN2* group was significantly decreased compared to that in the Vector group. (**l**) MFN2 was overexpressed in the HTH83 cell line, and images representing N-cadherin and E-cadherin expression are shown. In the control group (HTH83-Vector group), N-cadherin was only expressed in the cytoplasm, and E-cadherin was not expressed. In contrast, the expression of E-cadherin in the cytoplasm was significantly increased in the HTH83-MFN2 group. The image was acquired at a magnification of ×400; the scale bar represents 50 μm. Asterisks (p < 0.05 [*], p < 0.01 [**], p < 0.001 [***]) indicate significant differences in the statistical analyses. Each data point represents the mean ± standard error of three independent experiments.

Next, we determined whether there were differences in EMT markers at the mRNA level in Cal62 cells after *MFN2* KO. The relative expression of an epithelial marker, *CDH1*, was significantly downregulated in MFN2-KO cells compared with that in the control Cal-62 cells (*p* < 0.001, Fig. 3a-i). Conversely, the expression of a mesenchymal marker, *CDH2*, was significantly upregulated in MFN2-KO Cal62 cells (*p* < 0.001, Fig. 3a-ii). We also evaluated the abundance of EMT markers after MFN2-KO in Cal62 cells (Fig. 3b). There was a slight complementary increase in Mitofusin-1 (MFN1) levels after MFN2 KO, but it was not significant (Fig. 3c-i,c-ii). Consistent with the mRNA results, the N-cadherin protein levels significantly increased after MFN2-KO in Cal62 cells (*p* < 0.001, Fig. 3b,c-iii). We also examined the protein levels of major transcription factors involved in EMT, such as SNAIL and ZEB1 (TCF8). The protein levels of SNAIL and ZEB1 significantly increased after MFN2 KO in Cal62 cells (*p* < 0.01, and *p* < 0.05, respectively; Fig. 3b,c-iv,c-v). We also confirmed the effects *MFN2* knock-down by siRNAs in HTH83 cells (Fig. 3d, p < 0.01, Fig. 3e-i). The level of Vimentin significantly increased in si*MFN2* cells compared with that in control HTH83 cells (*p* < 0.01, Fig. 3e-ii). The level of E-cadherin, an epithelial marker, was suppressed (*p* < 0.05, Fig. 3e-iii) and the level of ZEB1 (TCF8), an EMT-related transcriptional factor, was induced (p < 0.01, Fig. 3e-iv) after *MFN2* knock-down in HTH83 cells, consistent with the data in Cal62 cells. These findings suggest that *MFN2* KO induces EMT in thyroid cancer cells.

To confirm the effect of *MFN2* on EMT, we evaluated the changes in the expression of EMT markers by overexpressing the gene in thyroid cancer cells. Consistent with previous data, the expression of *CDH1* mRNA was significantly induced, while that of *CDH2* mRNA was suppressed in *MFN2*-overexpressing Cal62 cells compared with that in the control cells (*p* < 0.001, and *p* < 0.001, respectively; Fig. 3f-i,f-ii). After overexpression of *MFN2*, there was a significant increase in MFN2 protein levels (*p* < 0.001, Fig. 3g,h-i) and no significant change in Mitofusin-1 protein levels (Fig. 3h-ii). The levels of the EMT-related transcription factors SNAIL and ZEB1 (TCF8) were significantly decreased upon *MFN2* overexpression in Cal62 cells (*p* < 0.01, and *p* < 0.001; Fig. 3h-iii,h-iv). Overexpression of *MFN2* in HTH83 cells resulted in increased expression of *CDH1* mRNA (p < 0.001, Fig. 3i-i), while that of *CDH2* mRNA decreased compared with that in the control cells (p < 0.001, Fig. 3i-ii). We evaluated the protein levels of EMT markers in HTH83 cells after overexpression of *MFN2* (Fig. 3j). There was a significant increase in MFN2 protein levels (Fig. 3k-i) and a complementary decrease in MFN1 levels after the overexpression of *MFN2* in HTH83 cells (Fig. 3k-ii). The expression of the epithelial marker E-cadherin was significantly increased after the overexpression of *MFN2* (*p* < 0.001, Fig. 3k-iii), and the expression of mesenchymal markers N-cadherin and Vimentin significantly decreased in *MFN2*-overexpressing cells compared with that in control HTH83 cells (*p* < 0.001 and *p* < 0.05, Fig. 3k-iv,k-v). In addition, the expression of EMT-related transcription factors SNAIL and ZEB1 (TCF8) also significantly decreased after *MFN2* overexpression in HTH83 cells (*p* < 0.01, Fig. 3k-vi, and *p* < 0.01, Fig. 3k-vii). These results suggest that MFN2 plays an important role as a negative regulator in the SNAIL- and ZEB1 (TCF8)-related EMT process in thyroid cancer cells.

We also confirmed the subcellular locations of E-cadherin and N-cadherin in *MFN2*-overexpressing cells and control HTH83 cells (Fig. 3l). The expression of E-cadherin was increased in the cytoplasm and cell membrane in *MFN2*-overexpressing cells compared with that in control cells. However, very low N-cadherin expression was detected in *MFN2*-overexpressing cells compared with that in the control group.

### Mitofusin-2 is a negative regulator of the PI3K/AKT signaling pathway

**Figure 4:**
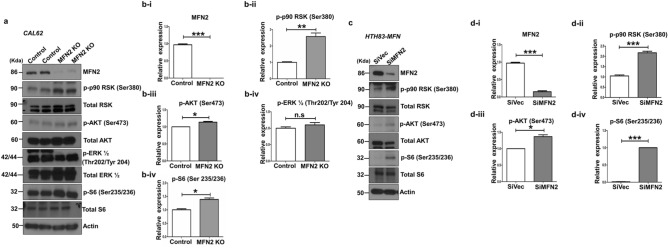
Mitofusin-2 is a negative regulator of the PI3K/AKT signaling pathway. (**a**) Immunoblotting of Mitofusin-2, *p-p*90 RSK (Ser380), *p*AKT (Ser473), *p*ERK1/2 (Thr202/Tyr204), and *p-*S6 (Ser 235/236) was conducted using cell lysates from *MFN2* KO and control Cal62 cells. (**b**) Quantification of immunoblotting for (**b-i**) *MFN2,* (B-ii) *p-p*90 RSK (Ser380), (**b-iii**) *p-AKT* (Ser473), (**b-iv**) *p*ERK1/2 (Thr202/Tyr204), and (**b-v**) *p-*S6 (Ser 235/236) after *MFN2* KO in Cal62 cells. (**c**) Immunoblotting for investigating the levels of MFN2, *p-p*90 RSK (Ser380), *p*AKT (Ser473), and *p-*S6 (Ser235/236) was performed using cell lysates from SiMFN2 HTH83 and control SiVector HTH83 cells. (**d**) Quantification of the levels of (**d-i**) *MFN2*, (**d-ii**) *p-p*90 RSK (Ser380), (**d-iii**), *p-AKT* (Ser473), and (**d-iv**) *p-*S6 (Ser 235/236) after transfection with SiMFN2 in HTH83 cells. Asterisks (p < 0.05 [*], p < 0.01 [**], p < 0.001 [***]) indicate significant differences from the statistical analyses. Each data point represents the mean ± standard error of three independent experiments.

To clarify the potential mechanism by which MFN2 acts as a tumor suppressor in *RAS*-mutated thyroid cancer cells, we also evaluated the changes in the PI3K-AKT pathway. PI3K pathway is a well-known downstream signaling pathway in *RAS* mutated cancer cells and there was a positive correlation of *MFN2* mRNA expression and *RAS* like gene expression in our data. Previous studies suggested that MFN2 interact with RAS or suppresses cancer progression through inhibition of this signaling pathway in other cancers^12–14^. First, we compared the changes in the levels of p-p90 RSK (Ser380), pAKT (Ser473), and pS6 (Ser235/236) between *MFN2* KO and control Cal62 cells (Fig. 4a,b). The levels of these phosphorylated proteins were significantly increased after KO of *MFN2* in Cal62 cells (Fig. 4a,b). However, p-ERK levels were not different between *MFN2* KO and control Cal62 cells (Fig. 4a). Second, following the significant decrease in MFN2 protein levels after *MFN2* knockdown by siRNA in HTH83 cells (Fig. 4c,d-i), significant increases in p-p90 RSK (Ser380), pAKT (Ser473), and pS6 (Ser235/236) levels were observed (Fig. 4c,d). These data suggest that MFN2 is a negative regulator of the PI3K-AKT signaling pathway in these thyroid cancer cells.

Conversely, we also investigated the molecular changes that occur in Cal62 and HTH83 cells when they overexpress *MFN2* (Supplementary Fig. 7). Following the significant increase in MFN2 protein expression after overexpression in both Cal62 and HTH83 cells (*p* < 0.01, Supplementary Fig. 7B-i,D-i), significant decreases in p-p90RSK (Ser380) and pS6 (Ser 235/236) levels were observed in Cal62 cells (*p* < 0.01 and *p* < 0.05, Supplementary Fig. 7A,B-ii,B-iv) and in p-p90RSK (Ser380) and pAKT (Ser473) levels in HTH83 cells (*p* < 0.01 and *p* < 0.06, Supplementary Fig. 7C,D-ii,D-iii).

### Overexpression of *MFN2* inhibits EMT in mouse xenograft models

**Figure 5:**
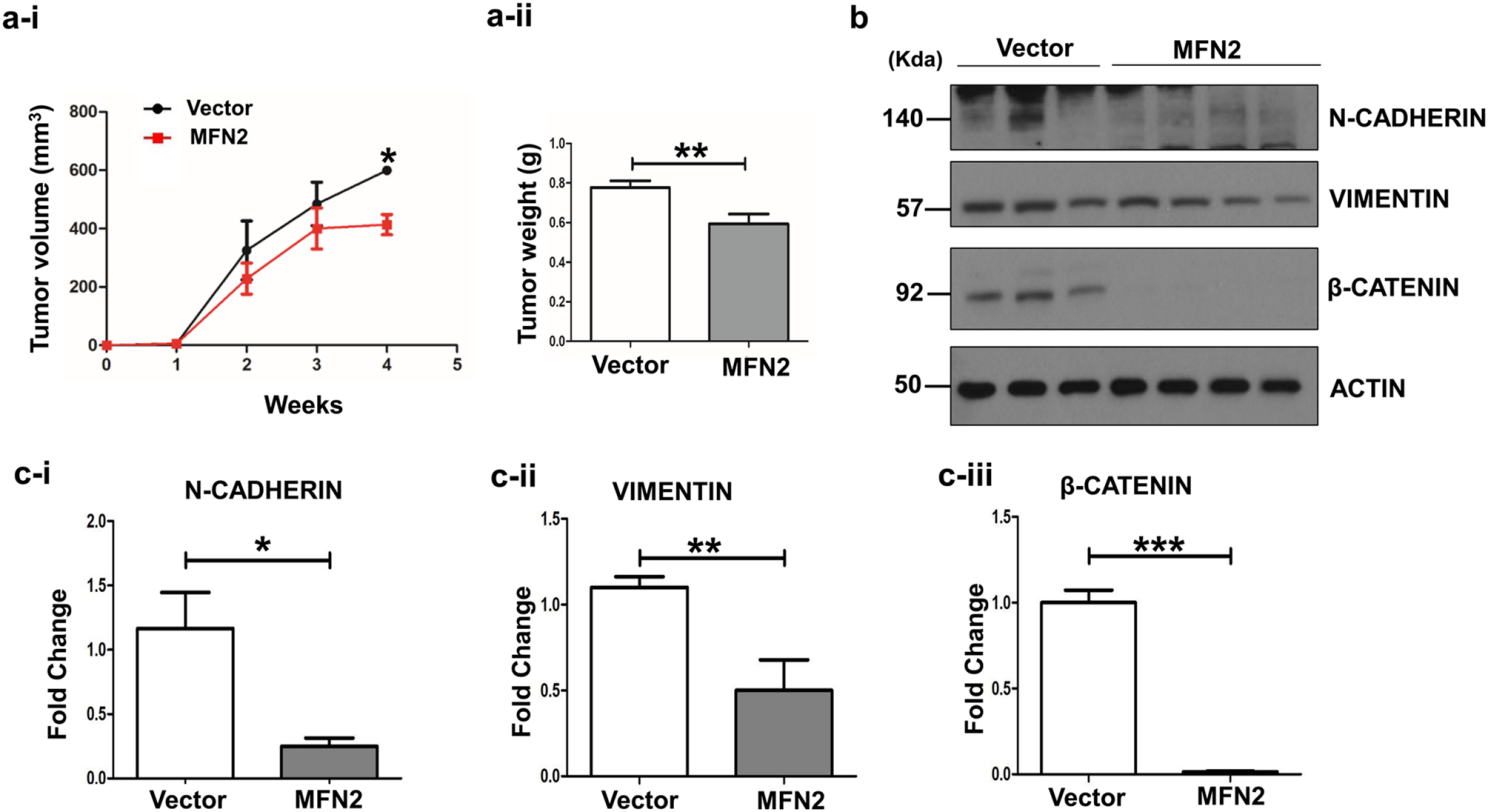
Tumor growth in a mouse xenograft model after gain of function of *MFN2*. The gain of function of *MFN2* in the Cal62 cell line was investigated in a subcutaneous xenograft model. (**a-i**) Graph of tumor growth to determine whether MFN2 overexpression inhibits tumor growth. (**a-ii**) Graph of tumor volume. (**b**) Immunoblotting of Vimentin, N-cadherin, and β-catenin using in vivo tumor samples. (**c-i**) Quantification of (**b**) Vimentin. (**c-ii**) Quantification of (**b**) N-cadherin. (**c-iii**) Quantification of (**b**) β-catenin. Asterisks (p < 0.05 [*], p < 0.01 [**], p < 0.001 [***]) indicate significant differences from the statistical analyses. Each data point represents the mean ± standard error of three independent experiments.

To clarify the role of MFN2 in vivo, a subcutaneous xenograft model of Cal62 cells was used. The tumor volume in the *MFN2-*overexpressing Cal62 group was significantly lower than that in the control vector Cal62 group (*p* < 0.05, Fig. 5a-i). The tumor weights at the study end-point were significantly lower in the *MFN2* overexpression group those in the control group (*p* < 0.01, Fig. 5a-ii).

We evaluated the changes in EMT-associated proteins (N-cadherin, Vimentin, and β-catenin) in xenograft tumors between the MFN2 overexpression group and control group using western blotting (Fig. 5b,c). The protein levels of N-cadherin, Vimentin, and β-catenin were significantly lower in *MFN2*-overexpressing tumors than those in the control Cal62 tumors (*p* < 0.05, Fig. 5c-i, p < 0.01, Fig. 5c-ii, and *p* < 0.001, Fig. 5c-iii, respectively). These in vivo results indicate that *MFN2* overexpression suppressed EMT not only in vitro but also in vivo.

## Discussion

In this study, we evaluated the potential role of MFN2 in thyroid cancer using TCGA data. The expression of *MFN2* was significantly decreased in tumor tissues compared with that in normal controls. PTCs with relatively higher *MFN2* expression were associated with well-differentiated tumors, *RAS* mutations, and less LN metastasis. The results of the gene set enrichment analysis suggest that there is a possible association between *MFN2* expression and the EMT process. Consequently, downregulation of *MFN2* using CRISPR/Cas9 or siRNA increased thyroid cancer cell invasion and migration by inducing EMT, and this was associated with activation of the AKT signaling pathway. *MFN*2 overexpression also significantly decreased thyroid cancer cell invasion and migration by inhibiting EMT. These changes in the expression of EMT markers after the modulation of *MFN2* were also confirmed in in vivo xenograft experiments. Our results suggest that MFN2 plays an important role as a tumor suppressor in thyroid cancers by regulating EMT.

Recently, several studies have reported that MFN2 plays a potential role in various malignancies, including hepatocellular, gastric, bladder, breast, and lung cancers^11–14,20–22^. In studies that analyzed the MFN2 mRNA and protein levels in human tissues in various types of cancer, the expression of MFN2 was found to be negatively associated with cancer progression^12,13,15,16,20–22^. Specifically, the low MFN2-expressing group was found to exhibit shorter cancer survival and poorer prognosis than the high MFN2-expressing group in breast cancer^13^. These results are consistent with the experimental data and the results of TCGA analysis of thyroid cancer obtained in our study.

EMT is a reversible cellular biological program used in embryogenesis and wound healing. During EMT, cell–cell adhesive junctions are weakened as cells transition to a mesenchymal cell morphology, and cells express mesenchymal markers^16,23–25^. EMT is regulated by multiple EMT-inducing transcription factors, such as the Twist, SNAIL, and ZEB1 (TCF8) families^25^. These transcription factors regulate the expression of one another, induce mesenchymal marker expression, repress epithelial marker expression, and regulate the expression of extracellular matrix molecules. EMT activation in cancer is essential to the progression to malignancy (e.g. metastasis)^25,26^. Thus, EMT has emerged as a specific indicator of high-grade malignancy, and has been identified as a central driver of poor clinical outcomes^25,26^. Recently, several studies have suggested that metabolic alterations in cancer can drive EMT^8,25,26^. As a component of the outer membrane of mitochondria, MFN1/MFN2 can participate in cancer metastasis and cellular reprogramming of cancer stem cells^24,27–29^. A recent study suggested that mitochondrial fusion through the miR200C-PGC1α (peroxisome proliferator activated receptor γ coactivator 1)-MFN1 pathway facilitates EMT during breast cancer progression^24^. These fused mitochondria also induced cancer stem cell progeny formation and increased glutathione synthesis. These findings indicate the possibility that MFN1/MFN2 is an important regulator of EMT in cancer progression^24^. The results of our study are consistent with those of this previous study. In our study, MFN2 modulated EMT in thyroid cancer by regulating the expression of EMT-related transcription factors at mRNA and protein levels. These findings were also consistent with TCGA data showing a negative association between MFN2 expression and LN metastasis in PTC. These findings provide the first evidence that MFN2 is a negative regulator of EMT in thyroid cancer.

Other possible mechanisms by which MFN2 affects carcinogenesis have been studied in liver cancer, gastric cancer, and bladder cancer^13,20–22^. MFN2 has been found to induce cell cycle arrest and mitochondrial-dependent cell death (apoptosis) in liver cancer, gastric cancer, and bladder cancer^20–22^. In breast and liver cancers, MFN2 was found to inhibit AKT/mTORC2 (mammalian target of rapamycin complex 2) signaling and act as a tumor suppressor^13,21^. The results of our study also suggest that MFN2 plays a role as a tumor suppressor in thyroid cancers by inhibiting AKT signaling. However, MFN2 might play a limited role in suppressing ERK signaling in thyroid cancer cells, and this phenomenon might explain why there were no significant changes in cancer cell proliferation following the modulation of *MFN2* expression. Cell proliferation was significantly increased by MFN2 KO in MEFs in this study, and this was associated with the significant activation of the ERK and AKT signaling pathways in previous studies^13,14^.

In this study, the effects of MFN2 on cell proliferation were minimal. However, MFN2 can inhibit cancer cell invasion and EMT in thyroid cancer cells. Several hallmarks of cancer has been established including sustaining proliferative signaling, resisting cell death, evading growth suppressors, inducing angiogenesis, enabling replicative immortality, and activating invasion and metastasis^30^. In our study, we tried to evaluate the role of MFN2 as a tumor suppressor in cell proliferation and cancer cell invasion in *RAS* mutated thyroid cancer cells because of a significant association of *MFN2* expression with *RAS* like phenotype in mRNA expression. MFN2 might play a role as a tumor suppressor on both proliferation and cell invasion in the other cancers^11,12,14^. What we found in this study is that MFN2 can inhibits cancer cell invasion through inhibiting EMT process and MFN2 has limited effects of cell proliferation and tumor growth in thyroid cancer cell model. In this study, we evaluated the role of MFN2 only using the *RAS* mutated thyroid cancer cells (Cal62 and HTH83) originated from anaplastic thyroid carcinoma. This can be one of the reasons for the limited effect on cell proliferation by MFN2 in this study. However, immortalized human cell lines from PTC, follicular carcinoma or anaplastic carcinoma have high proliferation rate than the in vivo differentiated thyroid tumors of patients. A recent comprehensive genetic profiling study also presented that gene expression profile of 58 thyroid cancer cell lines are profoundly dedifferentiated as demonstrated by low TDS comparable to that of human ATC specimens regardless of their derivation^18^. The dedifferentiation likely occurs when tumor cells adapt to the in vitro growth conditions^18^. Currently, no thyroid cancer cell line has similar differentiation status with human PTC. However, these cell lines still show dependence on their driver mutations for their biology and viability^18^. Therefore, we conducted functional study using thyroid cancer cells with *RAS* mutation (*KRAS* mutation in Cal62 and *HRAS* mutation in HTH83) because high MFN2 expressed PTC are related with *RAS*-like gene expression. As shown in the MEF cell experiment (Supplementary Fig. 4), MFN2 might suppress cell proliferation in the other cancer cells. The effects of tumor suppressor genes might be different depending on the type of cell or the genetic variation of the cells.

In conclusion, MFN2 was found to play an important role as a tumor suppressor in thyroid cancer progression and metastasis through modulating EMT and AKT signaling pathway. MFN2 might be an important prognostic marker of thyroid cancer and therapeutic target for its treatment based on its significant association with prognosis and LN metastasis in thyroid cancer.

## Supplementary Information

Supplementary Information.
