# Supplementary material for: Mitofusin-2 modulates the epithelial to mesenchymal transition in thyroid cancer progression
Source: Sci Rep. 2021 Jan 21;11:2054. doi: 10.1038/s41598-021-81469-0 (PMC7820342; doi:10.1038/s41598-021-81469-0)

Mitofusin-2 modulates the epithelial to mesenchymal transition in thyroid cancer progression

Mi-Hyeon You[1], Min Ji Jeon[1], Seong ryeong Kim[2], Woo Kyung Lee[3], Sheue-yann Cheng[3], Goo Jang[2], Tae Yong Kim[1], Won Bae Kim[1], Young Kee Shong[1], Won Gu Kim[1]

[1]Department of Internal Medicine, Asan Medical Center, University of Ulsan College of Medicine, Seoul, Korea

[2]College of Veterinary Medicine, Seoul National University

[3] Laboratory of Molecular Biology, Center for Cancer Research, National Cancer Institute, National Institutes of Health, Bethesda, Maryland 20892-4264, USA

Running title: Mitofusin-2 deficiency elicits EMT in thyroid cancer

Keywords: Mitofusin-2, papillary thyroid cancer, EMT (epithelial to mesenchymal transition), tumor suppressor, Ras mutation

Funding: This study was supported by a National Research Foundation of Korea Research Grant (NRF-2017R1D1A1B0303248 and NRF-2018R1D1A1A02085365).

Mi-Hyeon You: mhyou80@gmail.com

Min Ji Jeon: mj080332@gmail.com

Seong ryeong Kim: sungryung92@naver.com

Woo Kyung Lee: wookyung.lee@nih.gov

Sheue-yann cheng: chengs@mail.nih.gov

Goo Jang: snujang@snu.ac.kr

Tae Yong Kim: tykim@amd.seoul.kr

Won Bae Kim: kimwb@amc.seoul.kr

Young Kee Shong: ykshong@amc.seoul.kr

Won Gu Kim: wongukim@amc.seoul.kr

Corresponding author and person to whom reprint requests should be addressed

Won Gu Kim, M.D., Ph.D.

Department of Internal Medicine, Asan Medical Center, University of Ulsan College of Medicine

88, Olympic-ro 43-gil, Songpa-gu, Seoul 05505, Korea

Phone: +82-2-3010-5883; Fax: +82-2-3010-6962; E-mail: wongukim@amc.seoul.kr

Disclosure statement: The authors have nothing to disclose

Supplementary Figure 1

MFN2 expression is a prognostic marker in human thyroid cancer.

(A-i) Relationship of *MFN2* expression with TDS. Correlation analysis using Pearson correlation coefficient and (A-ii) comparison of *MFN2* expression between high and low TDS tumors.

(B-i) Relationship of *MFN2* expression with BRS. Correlation analysis using Pearson correlation coefficient and (B-ii) comparison of *MFN2* expression between high and low BRS tumors.

(C) Comparison of MFN2 expression between tumors without and with LNM. Data represent the mean ± SD. Asterisks (P < 0.001 [***], P < 0.0001 [****]) indicate significant differences from the statistical analyses. Abbreviations: TDS, thyroid differentiation scores; BRS, BRAFV600E-RAS scores; LNM, lymph node metastasis.

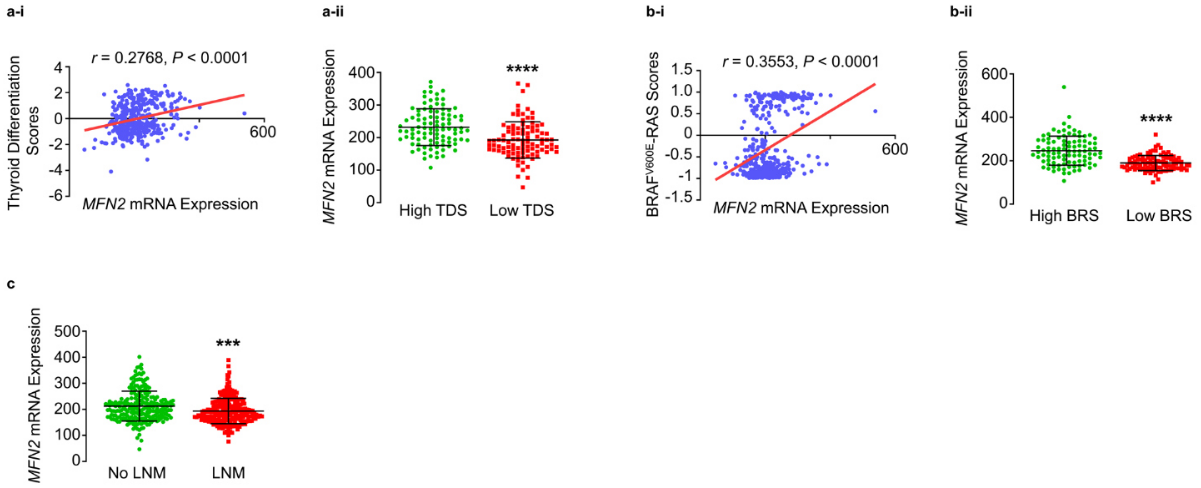

Supplementary Figure 2

(A) Knockout of MFN2 in the Cal 62 cell line. (A-i) Information on two guide RNAs on exon 4 and exon 5. Mutation assay with the T7E1 enzyme on exon 4 (A-ii) and exon 5 (A-iii); M: marker DNA ladder, (1): negative control, 1: wild-type genome, 2: transfected cells, (+): positive control. (A-iv) MFN2 knockout in the Cal62 cell line confirmed by western blotting.

(B-i) Illustration of the MFN2 and GFP expression vector.

(B-ii) Representative GFP-positive colony (left: brightness, right: fluorescence).

(B-iii) MFN2 overexpression in the Cal62 and HTH83 cell lines confirmed by western blotting.

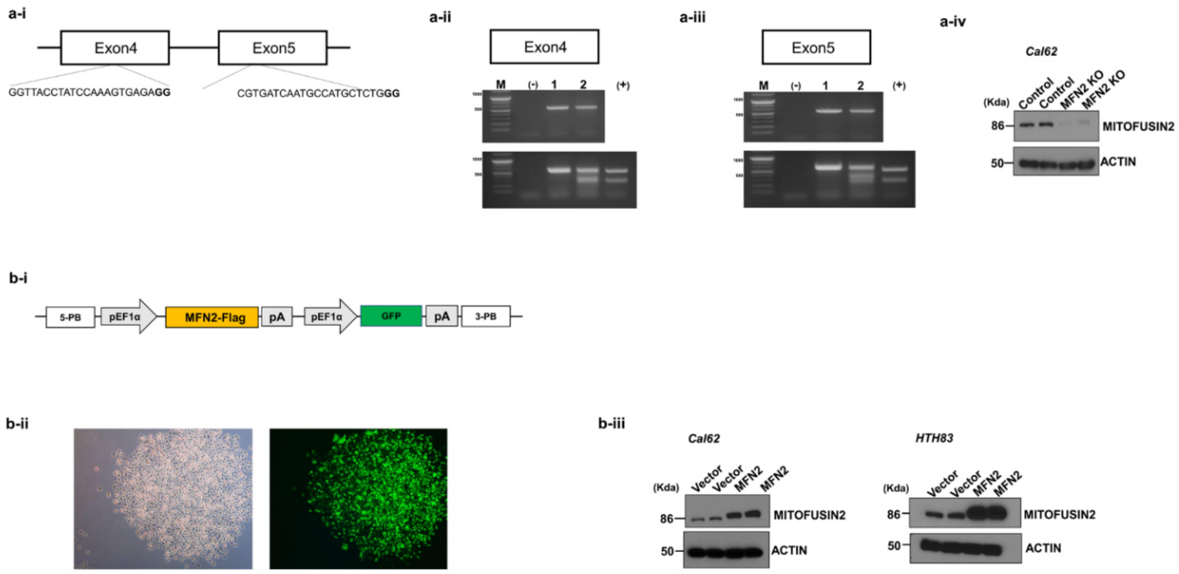

Supplementary Figure 3

(A) Mitofusin-2 was not associated with the proliferation ability in Cal62 cells.

(A-i) Assay for comparing the proliferation ability of *MFN2* KO and control Cal62 cells (n.s; not significant).

(A-ii) Assay for comparing colony formation between *MFN2* KO and control Cal62 cells on day 7. (A-iii) Quantification of the colony forming assay (n.s; no significant difference).

(A-iv) Assay for comparing the proliferation ability of Cal62 Vector and *MFN2* groups.

(A-v) Image representing the colony formation by the Cal62 Vector and *MFN2* groups on day 7. (A-vi) Quantification of (A-v).

(B) Mitofusin-2 was also not associated with cell proliferation ability in HTH83 cells.

(B-i) Assay for comparing the proliferation ability of HTH83 *MFN2*-SiVector and SiMFN2 groups (n.s; not significant).

(B-ii) Image representing colony formation by the HTH83 *MFN2*-SiVector and SiMFN2 groups. (B-iii) Quantification of (B-ii) (n.s; no significant difference).

(B-iv) Assay for comparing the proliferation ability of the HTH83 Vector and *MFN2* groups. (B-v) Image representing colony formation by the HTH83 Vector and *MFN2* groups.

(B-vi) Quantification of (B-v) (n.s; no significant difference). Asterisks (P < 0.01 [**]) indicate significant differences from the statistical analyses. Each data point represents the mean±standard error of three independent experiments.

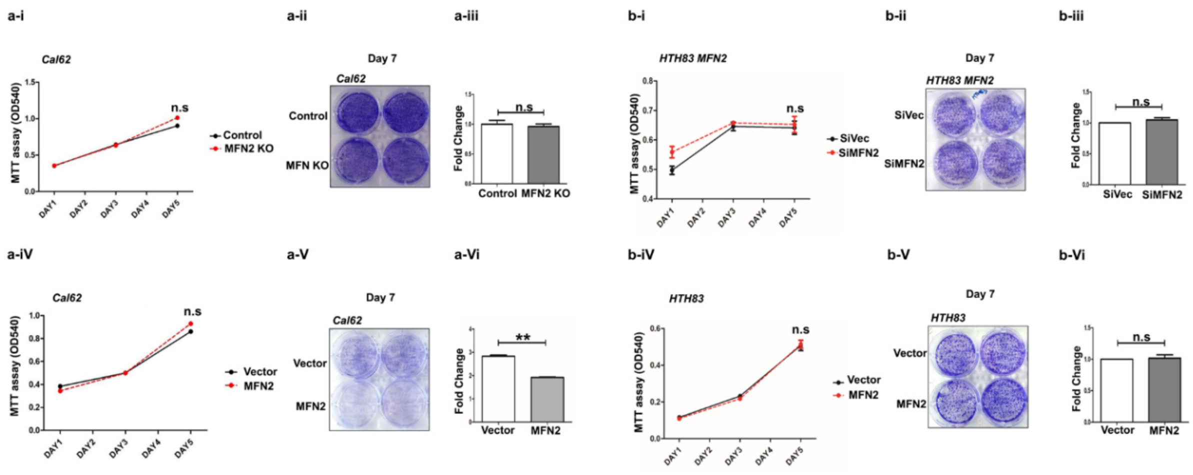

Supplementary Figure 4

(A) Mitofusin-2 was associated with proliferation ability of MEF cells. Assay for comparing the proliferation ability of the *MFN2* KO and MEF WT cells.

(B-i) Colony forming assay of *MFN2* KO and WT MEF cells on day 7.

(B-ii) Quantification of the colony forming assay. Asterisks (P < 0.05 [*], P < 0.01 [**], P < 0.001 [***]) indicate significant differences from the statistical analyses. Each data point represents the mean±standard error of three independent experiments.

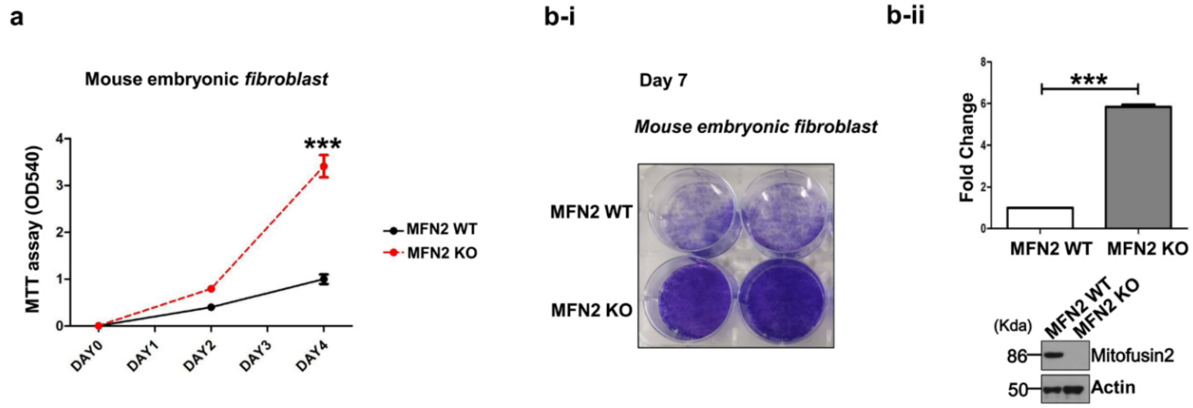

Supplementary Figure 5

(A-i) Transwell assays were performed in the HTH83 Vector and *MFN2* overexpression groups. A representative image was taken 12 h after the initial seeding (pore size 0.8 μm). (A-ii) Graph of the quantified results of (A-i).

(B-i) Wound-healing assays were performed to investigate the differences between the HTH83 Vector and HTH83 *MFN2* OE groups. We measured the gap distance 9 h after the initial scratching. (B-ii) Graph of the quantified results of (B-i). Asterisks (P < 0.05 [*], P < 0.01 [**], P < 0.001 [***]) indicate significant differences from the statistical analyses. Each data point represents the mean

±standard error of three independent experiments.

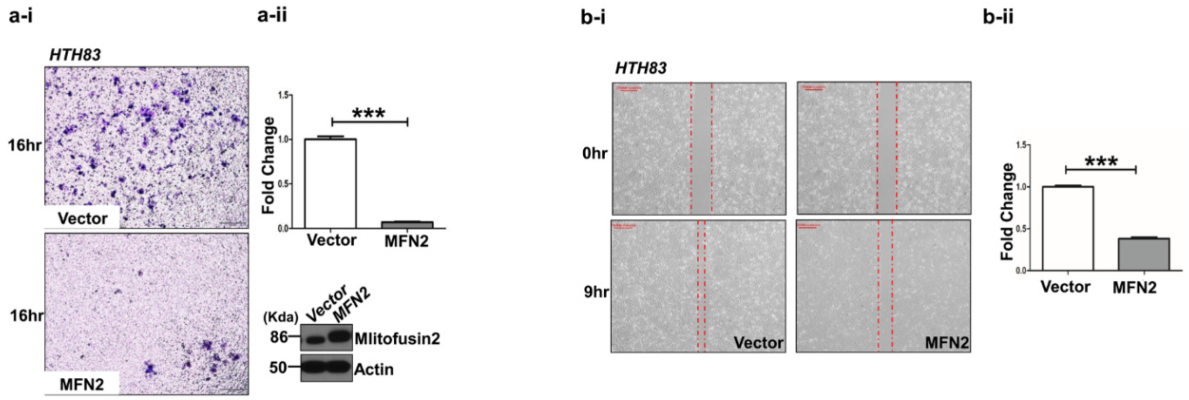

Supplementary Figure 6

(A-i) Transwell assays were performed in *MFN2* WT and KO MEF cells. A representative image was taken 24 h after the initial seeding (pore size 0.8 μm). (A-ii) Graph of the quantified results of (A-i). Significantly more cells migrated from *MFN2* KO MEFs than from the *MFN2* WT MEFs. (B-i) Wound-healing assays were performed to investigate the differences between *MFN2* WT and KO MEFs. We measured the gap distance 16 h after the initial scratching. (B-ii) Quantification of (B-i) compared to the initial scratch size of the first gap distance after 16 h. Asterisks (P < 0.05 [*], P < 0.01 [**], P < 0.001 [***]) indicate significant differences from the statistical analyses. Each data point represents the mean±standard error of three independent experiments.

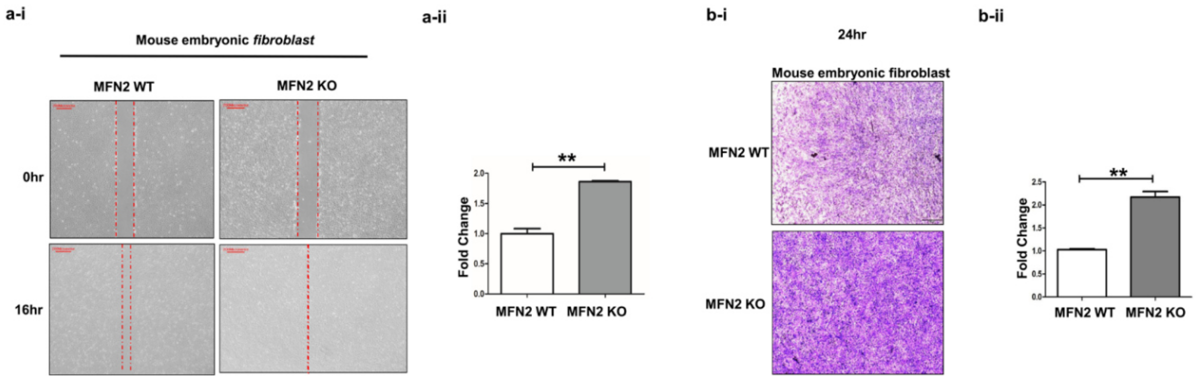

Supplementary Figure 7

MFN2 overexpression leads to pERK signal suppression.

(A) Immunoblotting of MFN2, *p-p*90 RSK (Ser380), *p*AKT (Ser473), and *p*-S6 (Ser235/236) was conducted using lysates from Cal62 C and Cal62 MFN2 OE cells.

(B) Quantification of (B-i) MFN, (B-ii) p-p90 RSK (Ser380), (B-iii) p-AKT (Ser473), and (B-iv) p-S6 (Ser235/236) levels.

(C) Immunoblotting for investigating the levels MFN2, *p-p*90 RSK (Ser380), *p*AKT (Ser473), and *p-* S6 (Ser235/236) was performed using cell lysates from the HTH83 Vector and HTH83 MFN2 overexpression groups.

(D) Quantification of (D-i) *MFN2*, (D-ii) *p-p*90 RSK (Ser380), (D-iii) *p-AKT* (Ser473), and (D-iv) *p*-S6 (Ser235/236). Asterisks (P < 0.05 [*], P < 0.01 [**], P < 0.001 [***]) indicate significant differences from the statistical analyses. Each data point represents the mean±standard error of three independent experiments.

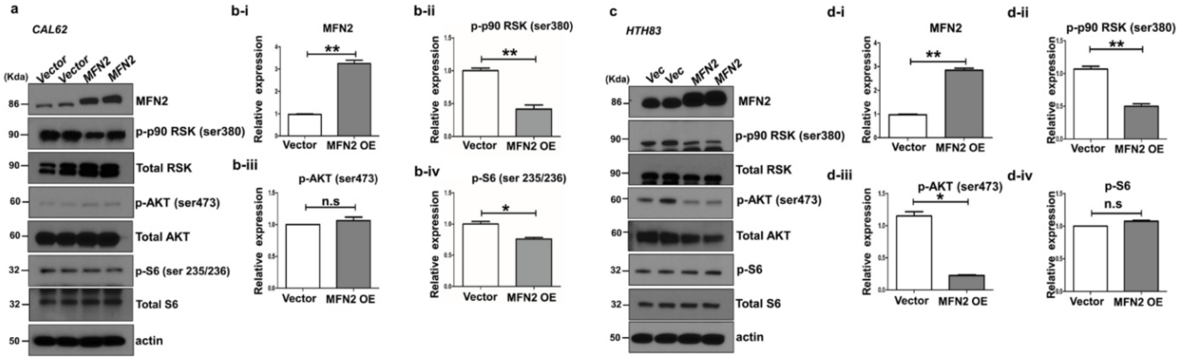

Supplementary Table 1. Real-time q-RT-PCR primer sequences

Supplementary Table 1. Primers used for mRNA expression analysis

Gene	Primer	5'→ 3' sequence
E-Cadherin	Forward	GAACGCATTGCCACATACAC
	Reverse	GAATTCGGGCTTGTTGTCAT
N-Cadherin	Forward	CCTGAGGGATCAAAGCCTGGAAC
	Reverse	TTGGAGCCTGAGACACGATTCTG
Snail	Forward	CTTCCAGCAGCCCTACGAC
	Reverse	CGGTGGGGTTGAGGATCT

Uncropped Gels shown as representative blots in Main& Supplementary Figures

Figure2

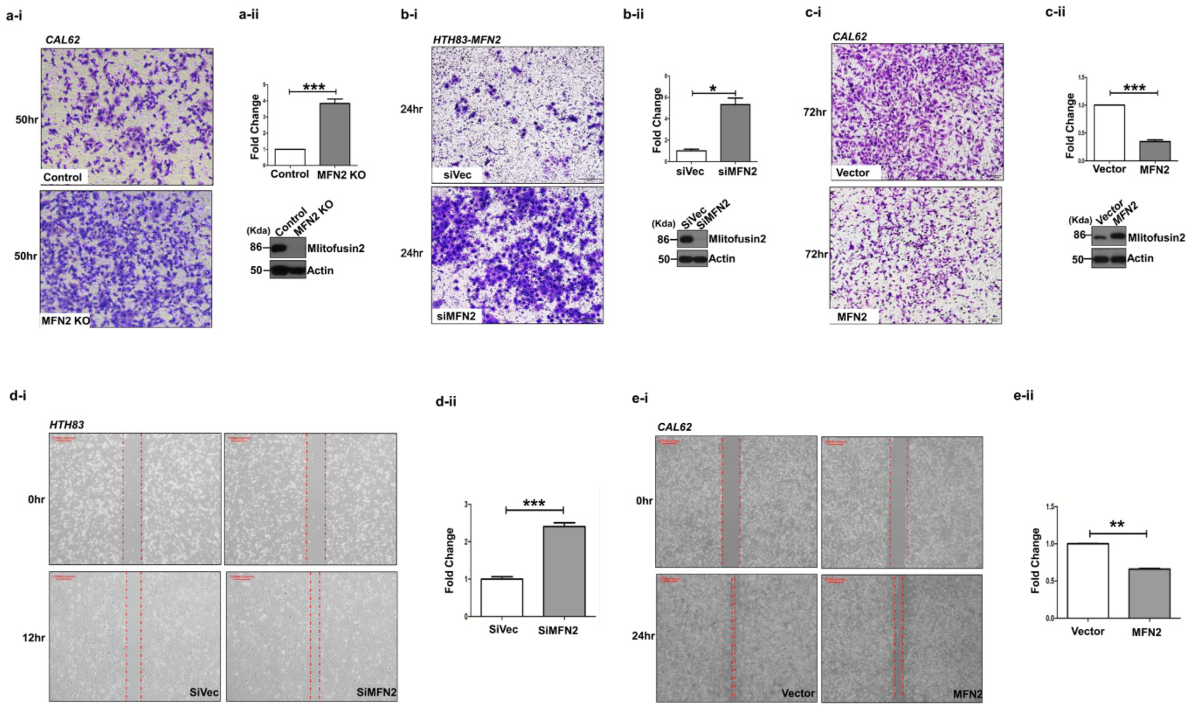

a-i
CAL62
50hr
Control
50hr
MFN2 KO
a-ii
Fold Change
***
Control
MFN2 KO
(Kda)
86
50
Mlitofusin2
Actin
b-i
HTH83-MFN2
24hr
siVec
24hr
siMFN2
b-ii
*
siVec
siMFN2
SiVec
SiMFN2
c-i
CAL62
72hr
Vector
72hr
MFN2
c-ii
Vector
MFN2
d-i
HTH83
0hr
12hr
SiVec
SiMFN2
d-ii
SiVec
SiMFN2
e-i
CAL62
0hr
24hr
Vector
MFN2
e-ii
**
Vector
MFN2

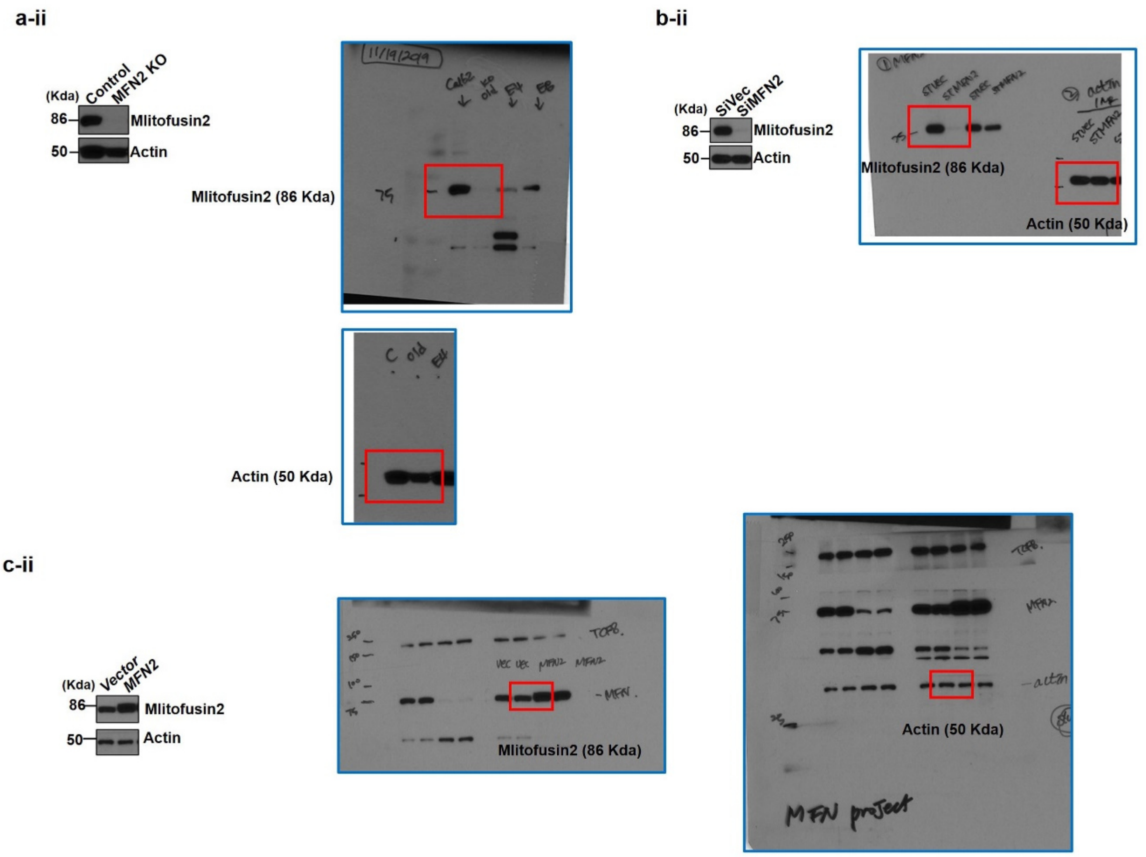

a-ii
(Kda)
Control
MFN2 KO
86
50
Mlitofusin2
Actin
Mlitofusin2 (86 Kda)
Actin (50 Kda)
b-ii
SiVec
SiMFN2
Mlitofusin2 (86 Kda)
Actin (50 Kda)
c-ii
Vector
MFN2
Mlitofusin2
Actin
Mlitofusin2 (86 Kda)
Actin (50 Kda)
MFN project

Figure3

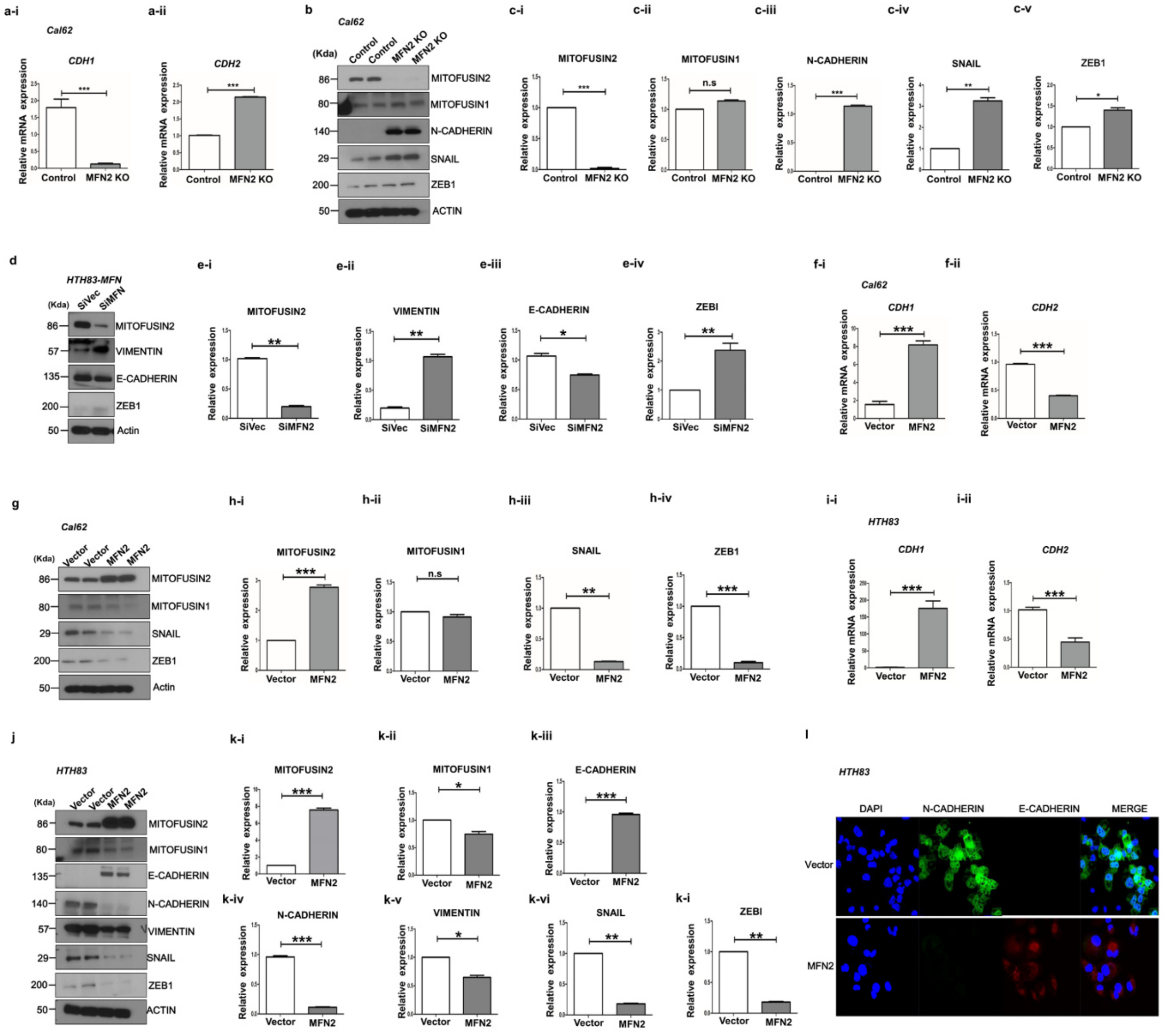

a-i
Cal62
CDH1
Relative mRNA expression
Control
MFN2 KO
a-ii
CDH2
b
Cal62
(Kda)
Control
MFN2 KO
MITOFUSIN2
MITOFUSIN1
N-CADHERIN
SNAIL
ZEB1
ACTIN
c-i
c-ii
c-iii
c-iv
c-v
Relative expression
n.s
d
HTH83-MFN
SiVec
SiMFN
VIMENTIN
E-CADHERIN
Actin
e-i
e-ii
e-iii
e-iv
SiMFN2
ZEBI
f-i
f-ii
Vector
MFN2
g
h-i
h-ii
h-iii
h-iv
i-i
i-ii
HTH83
j
k-i
k-ii
k-iii
k-iv
k-v
k-vi
l
DAPI
MERGE

b

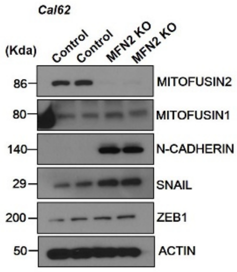
Cal62
(Kda)
Control
Control
MFN2 KO
MFN2 KO
86
MITOFUSIN2
80
MITOFUSIN1
140
N-CADHERIN
29
SNAIL
200
ZEB1
50
ACTIN

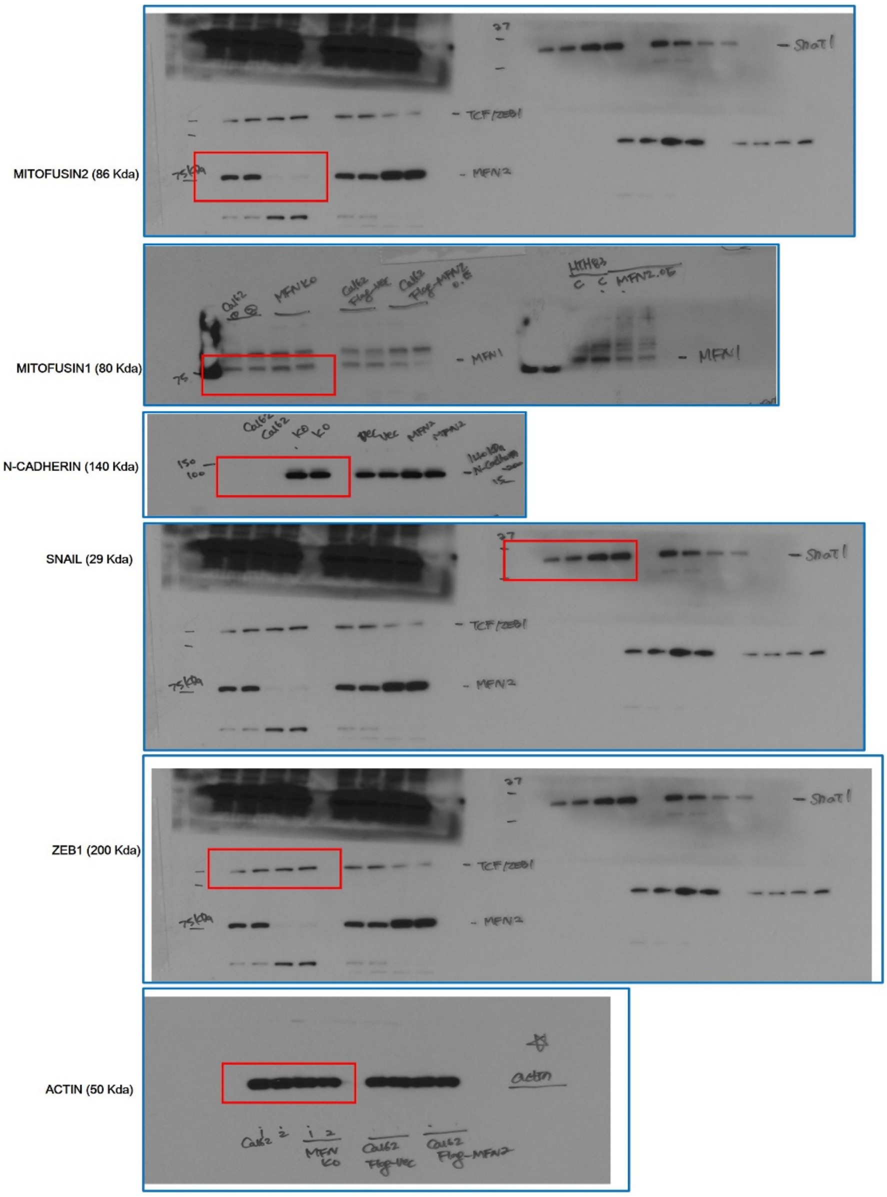
MITOFUSIN2 (86 Kda)
MITOFUSIN1 (80 Kda)
N-CADHERIN (140 Kda)
SNAIL (29 Kda)
ZEB1 (200 Kda)
ACTIN (50 Kda)

d

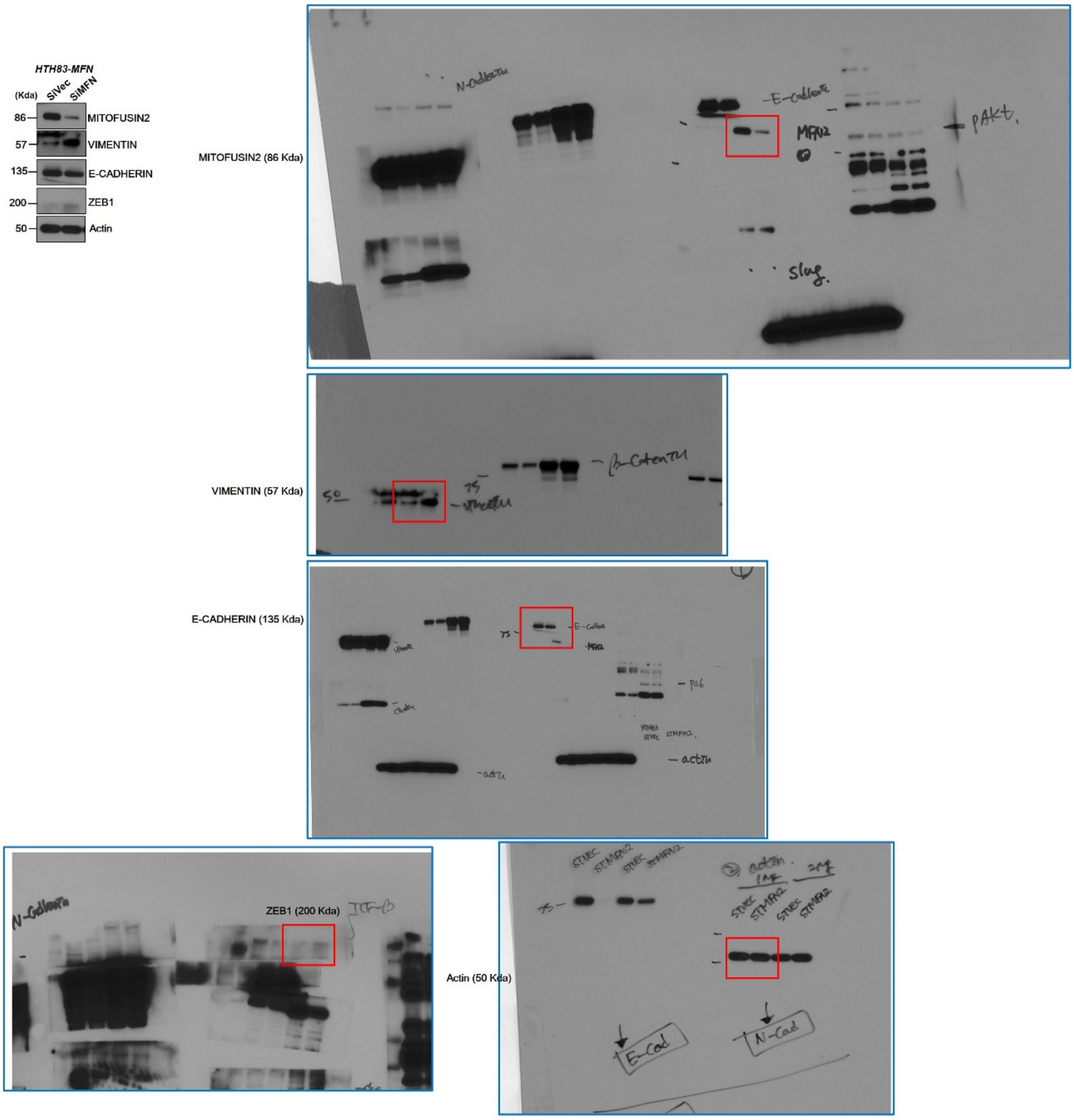
HTH83-MFN
SiVec
SiMFN
(Kda)
86
57
135
200
50
MITOFUSIN2
VIMENTIN
E-CADHERIN
ZEB1
Actin
MITOFUSIN2 (86 Kda)
VIMENTIN (57 Kda)
E-CADHERIN (135 Kda)
ZEB1 (200 Kda)
Actin (50 Kda)

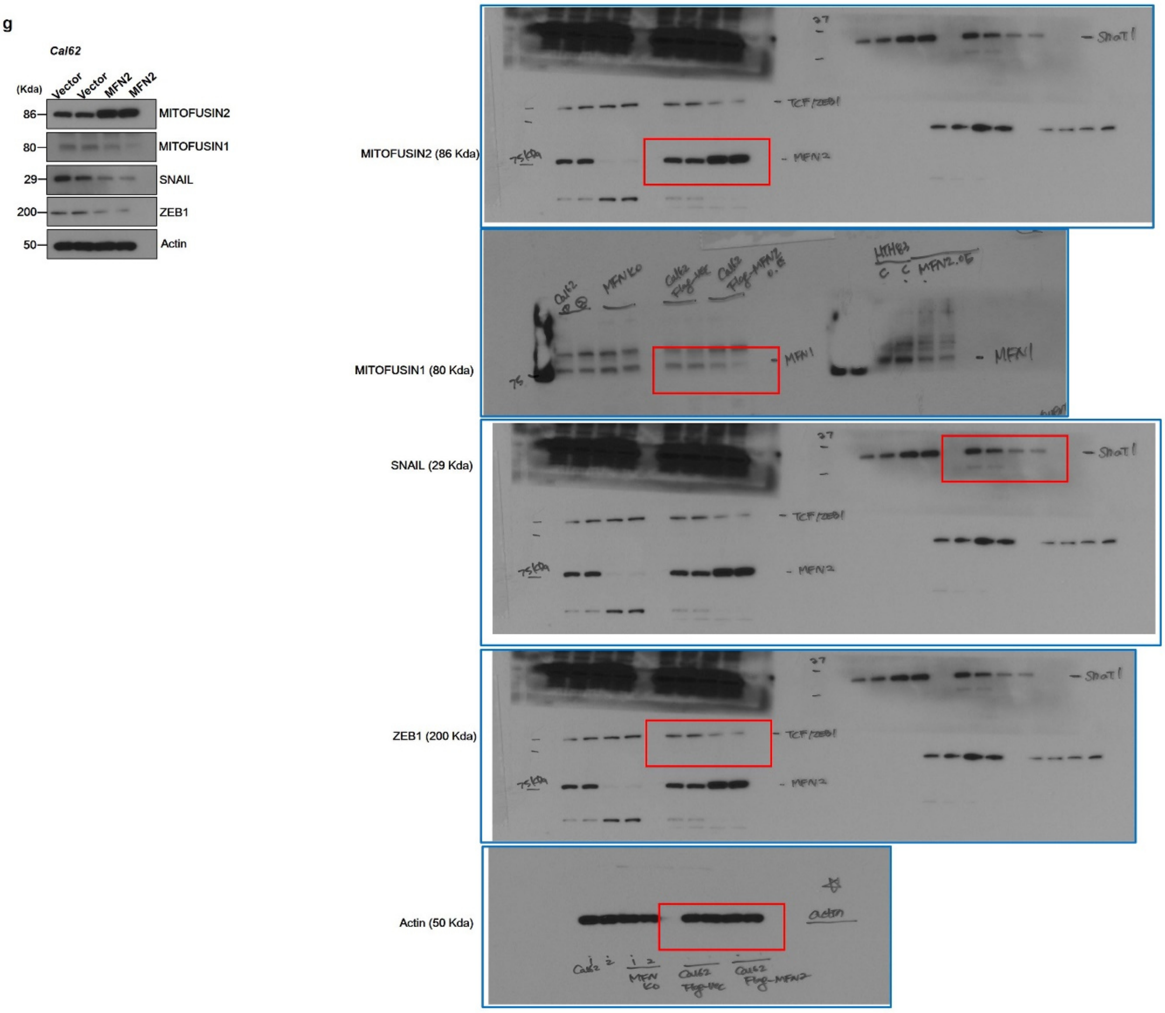
g
Cal62
(Kda)
Vector
Vector
MFN2
MFN2
86
80
29
200
50
MITOFUSIN2
MITOFUSIN1
SNAIL
ZEB1
Actin
MITOFUSIN2 (86 Kda)
MITOFUSIN1 (80 Kda)
SNAIL (29 Kda)
ZEB1 (200 Kda)
Actin (50 Kda)

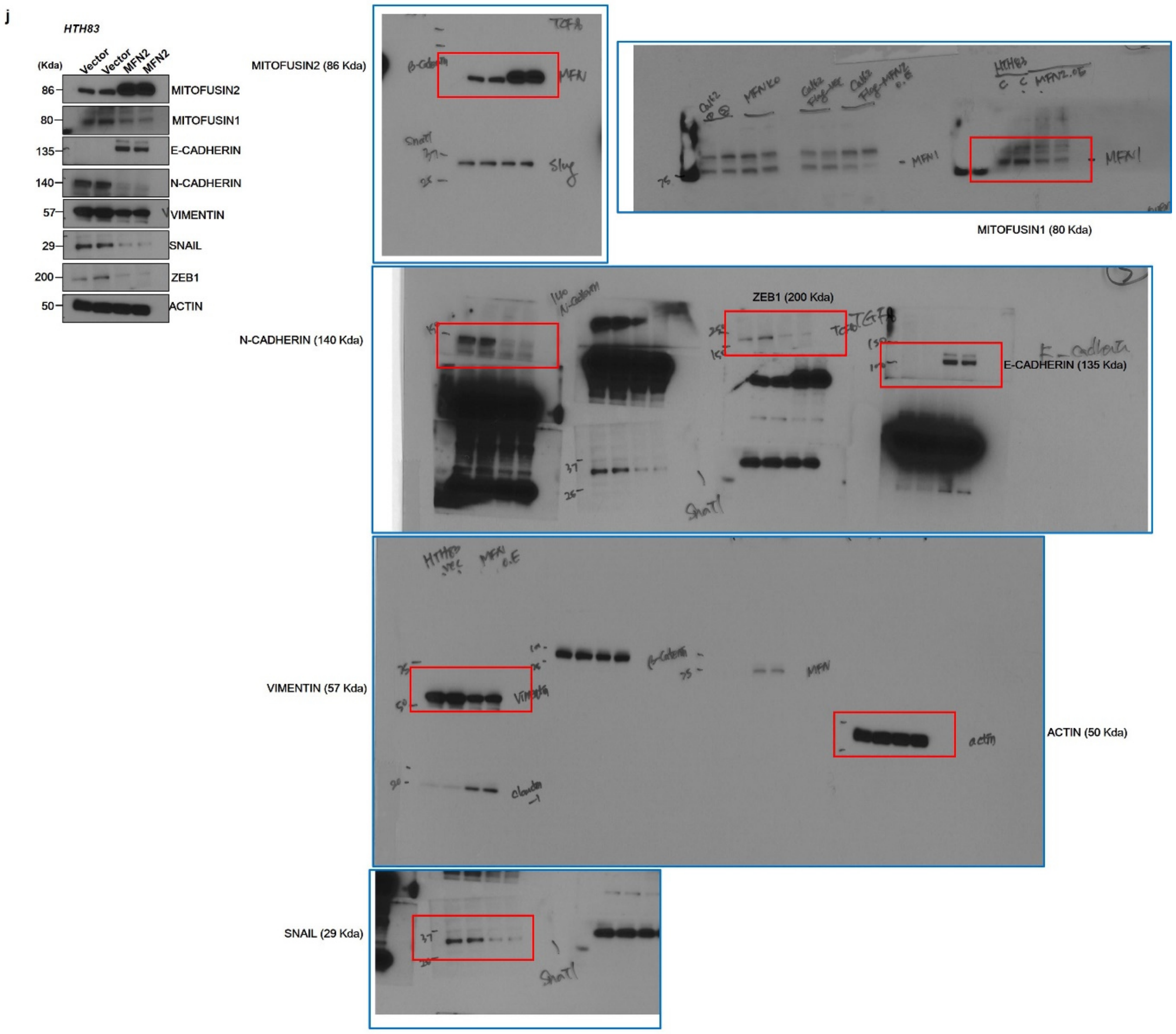
j
HTH83
(Kda)
Vector
Vector
MFN2
MFN2
86
MITOFUSIN2
80
MITOFUSIN1
135
E-CADHERIN
140
N-CADHERIN
57
VIMENTIN
29
SNAIL
200
ZEB1
50
ACTIN
MITOFUSIN2 (86 Kda)
MITOFUSIN1 (80 Kda)
N-CADHERIN (140 Kda)
ZEB1 (200 Kda)
E-CADHERIN (135 Kda)
VIMENTIN (57 Kda)
ACTIN (50 Kda)
SNAIL (29 Kda)

Figure 4

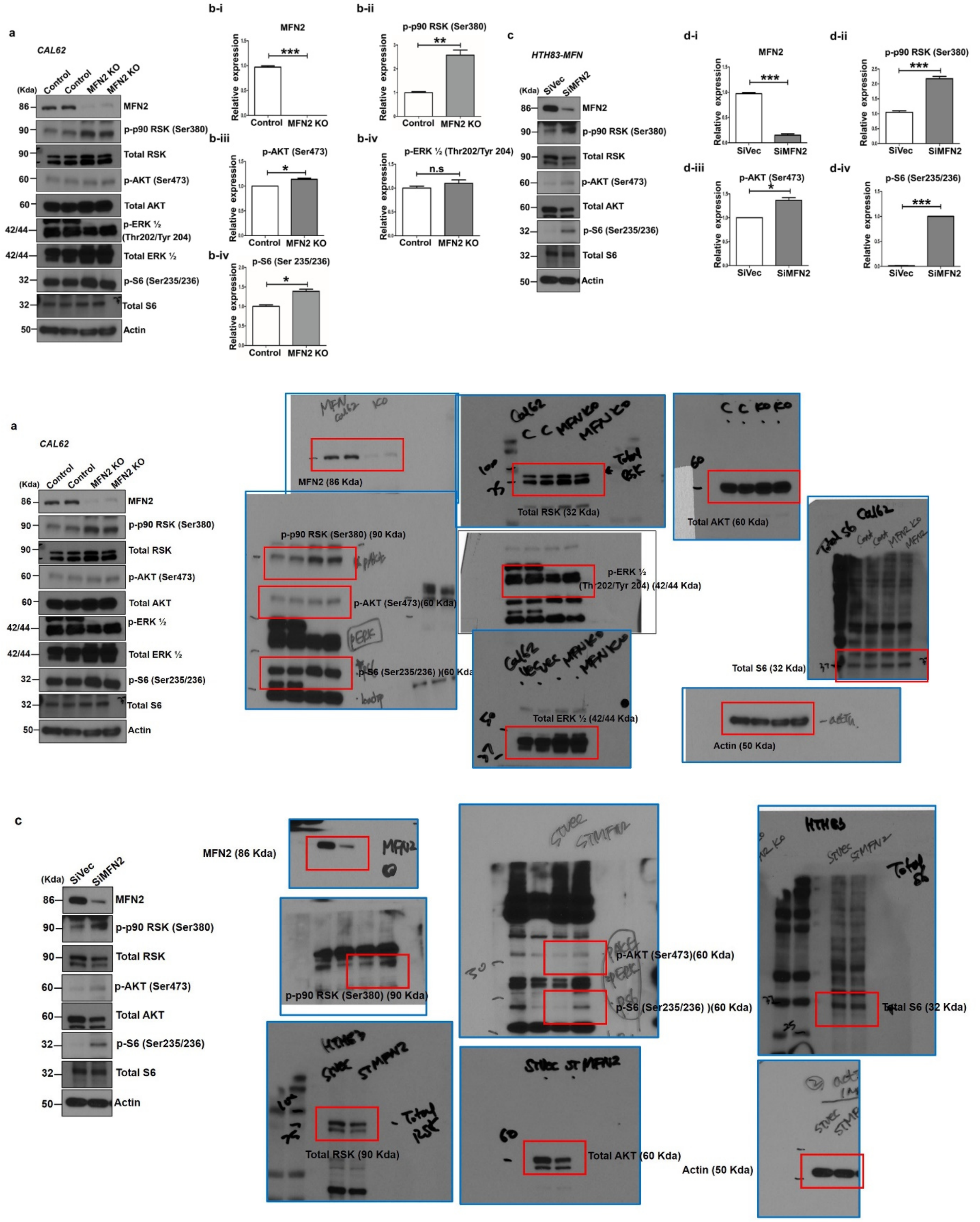

Figure 5

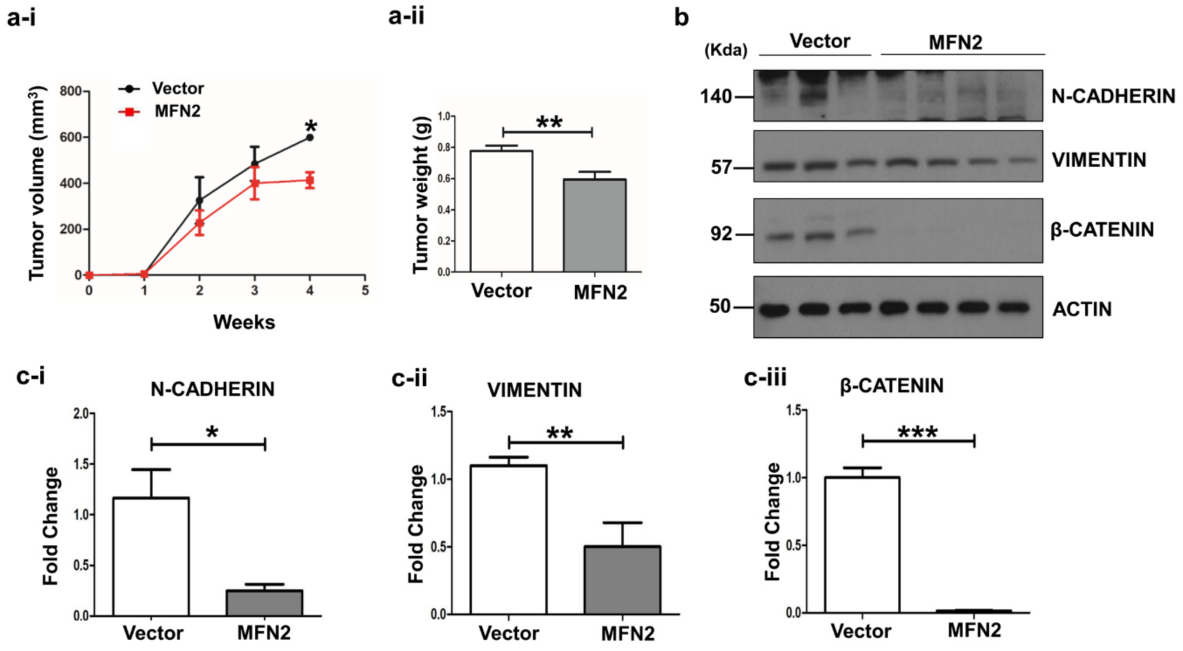
a-i
a-ii
b
Vector
MFN2
Tumor volume (mm³)
Weeks
Tumor weight (g)
(Kda)
140
57
92
50
N-CADHERIN
VIMENTIN
β-CATENIN
ACTIN
c-i
c-ii
c-iii
Fold Change

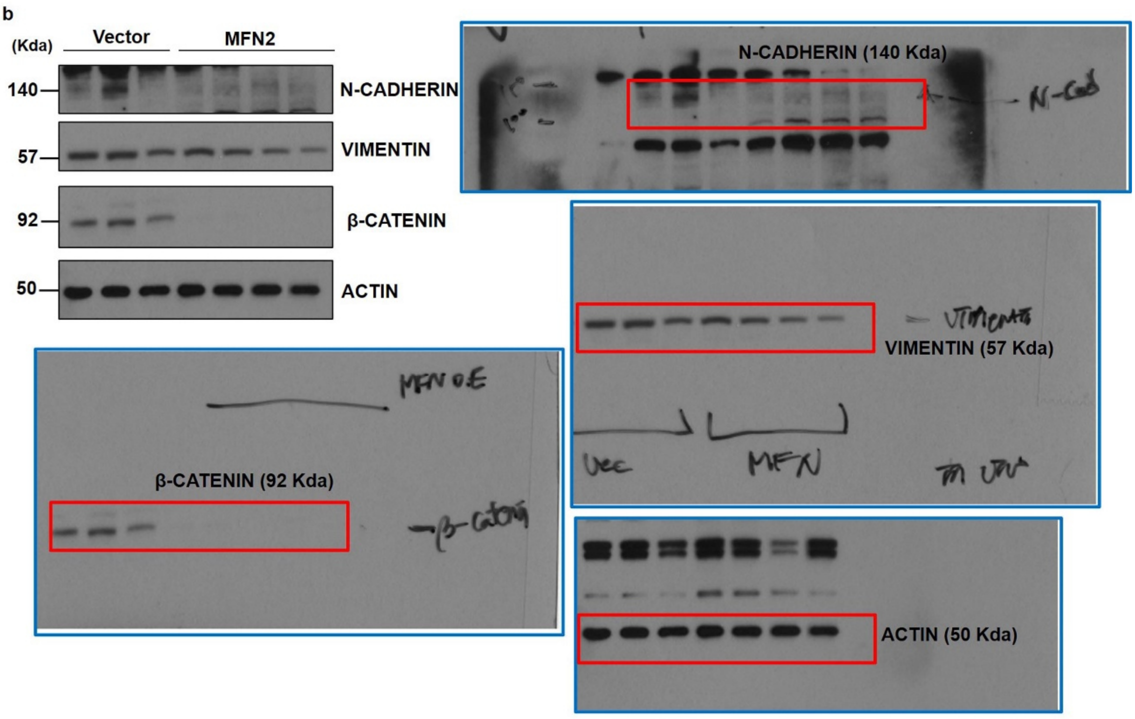
b
(Kda)
Vector
MFN2
N-CADHERIN
VIMENTIN
β-CATENIN
ACTIN
N-CADHERIN (140 Kda)
VIMENTIN (57 Kda)
β-CATENIN (92 Kda)
ACTIN (50 Kda)

Supplementary Figure 2

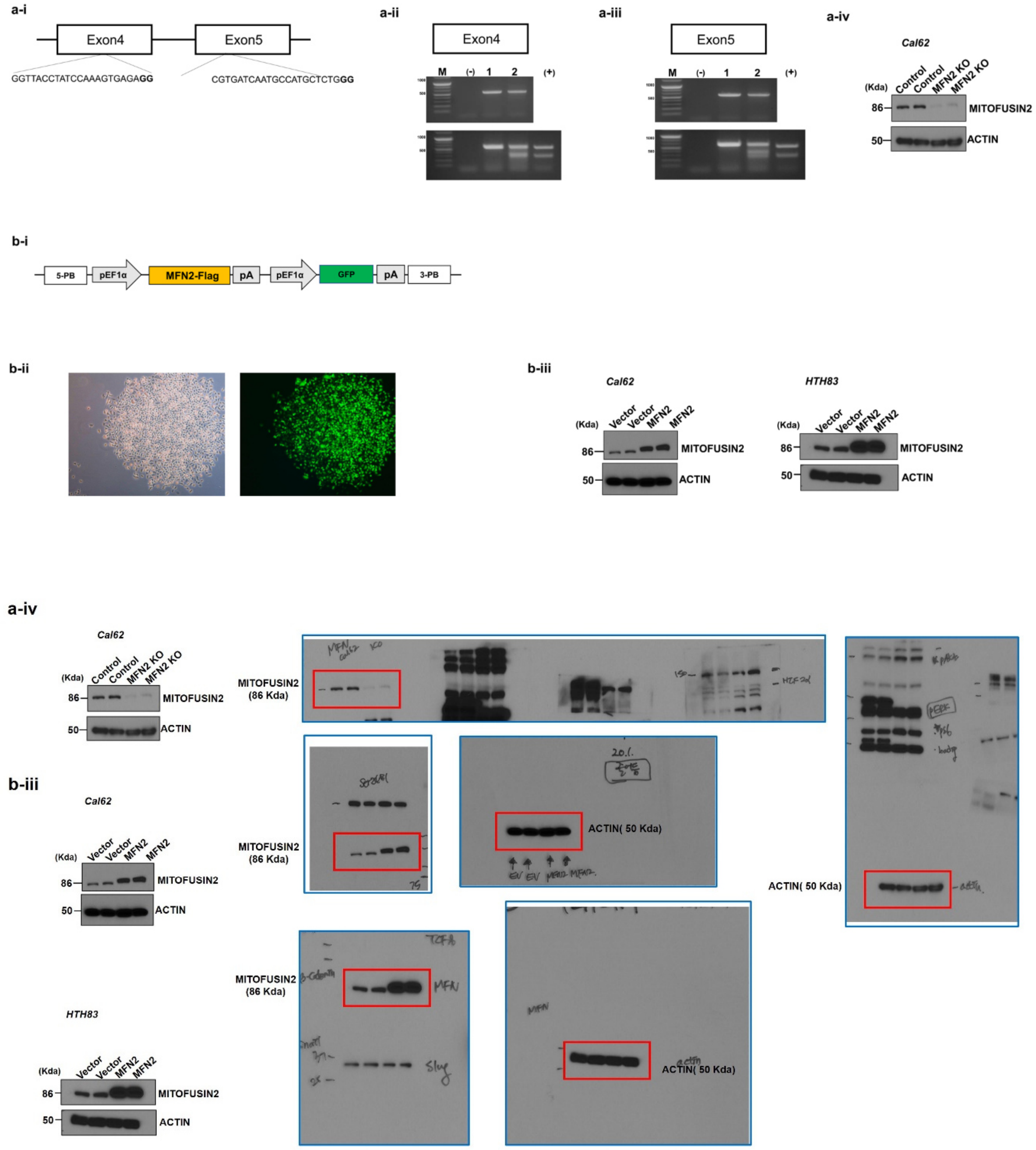

Supplementary Figure 4

a

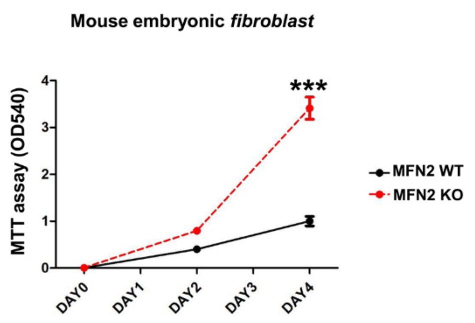

b-i

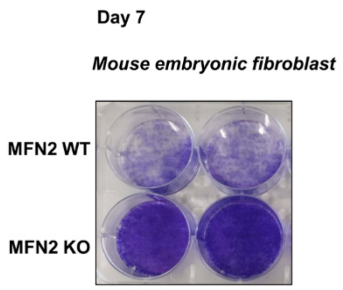

b-ii

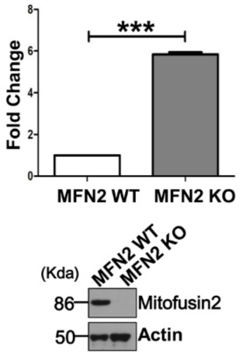

b-ii

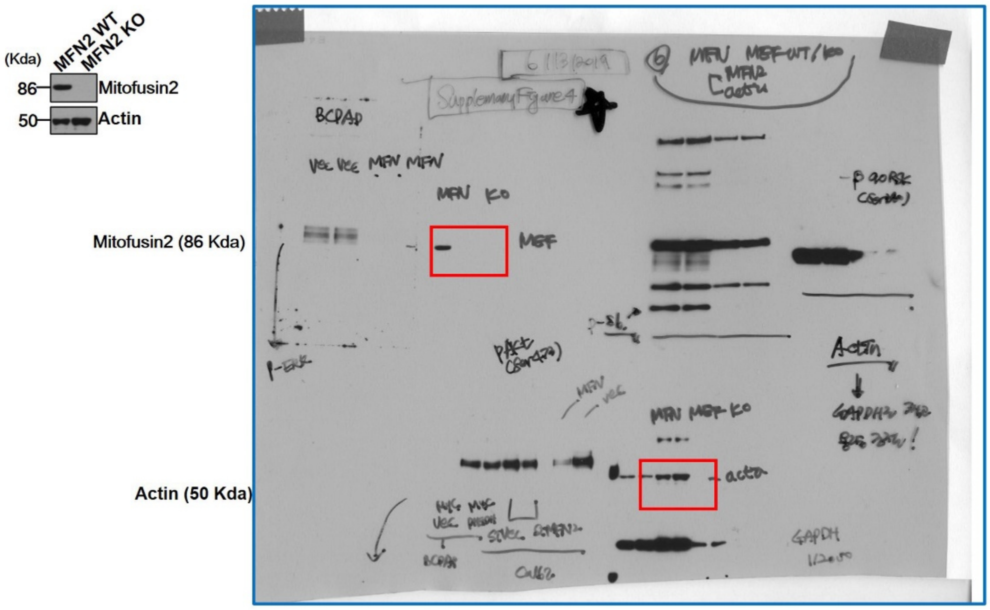

Supplementary Figure 5

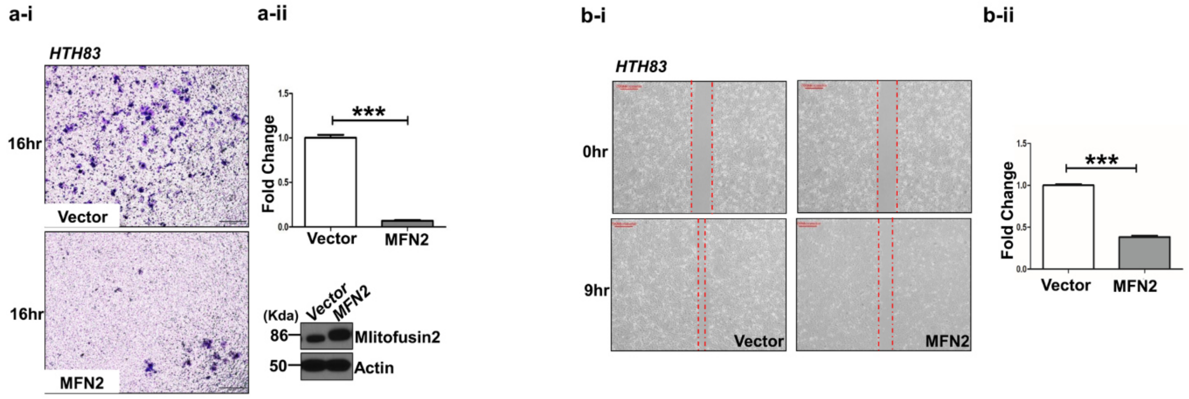

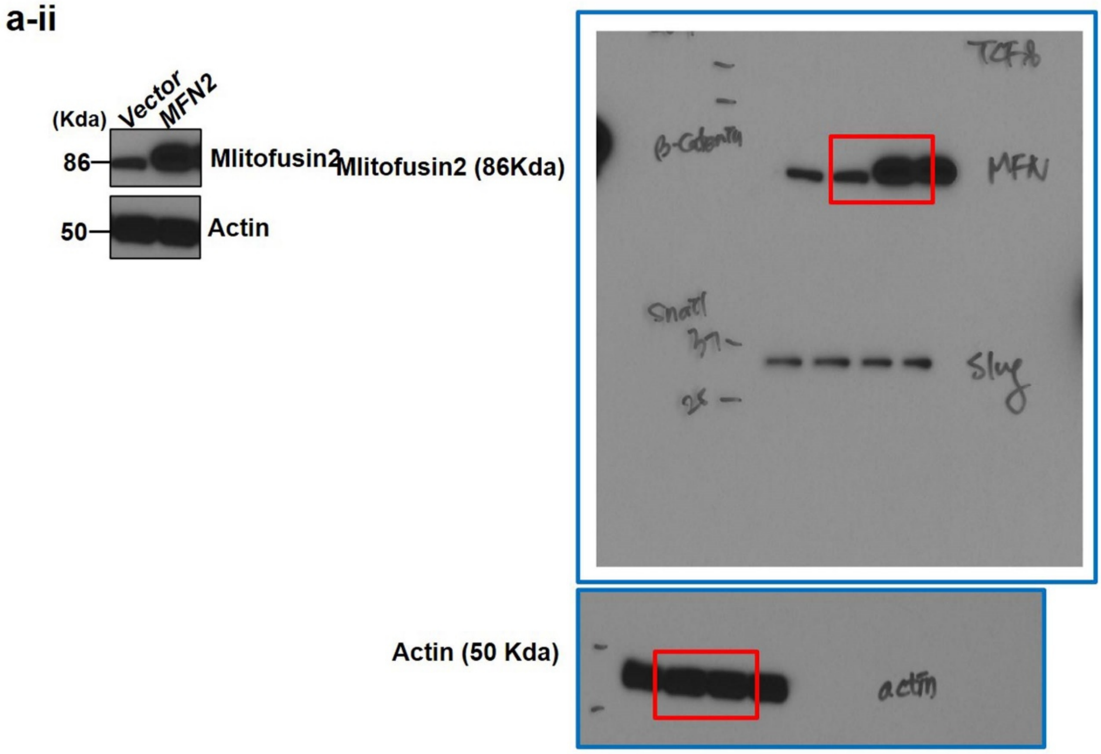

Supplementary Figure 7

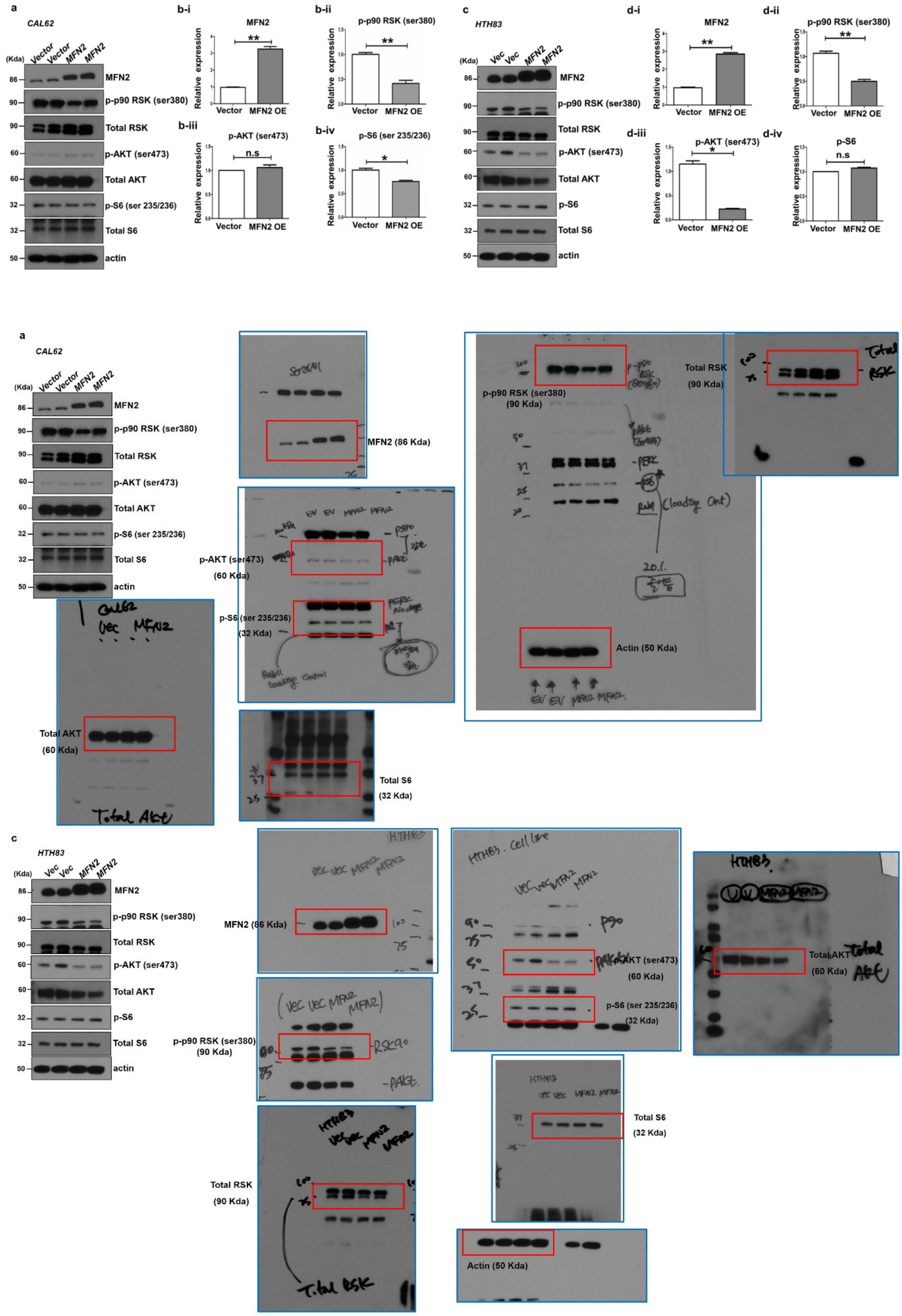

Supplement: Supplementary file 1 — Supplementary Information. [file 41598_2021_81469_MOESM1_ESM.pdf]
